# Reproductive aging in biological females: mechanisms and immediate consequences

**DOI:** 10.3389/fendo.2025.1658592

**Published:** 2025-09-12

**Authors:** Yasin Ali Muhammad

**Affiliations:** Department of Biology, Georgia State University, Atlanta, GA, United States

**Keywords:** aging, endocrine system, menopause (estrogen withdrawal), ovarian follicle cells, hypothalamas

## Abstract

Reproductive aging is a dynamic, systemic process that encompasses more than the decline in ovarian function. It involves coordinated changes across neuroendocrine, immune, metabolic, and mitochondrial systems. Central to this transition is the depletion of ovarian follicles, leading to reduced estradiol and progesterone production and subsequent disruption of the hypothalamic-pituitary-gonadal (HPG) axis. This hormonal shift remodels hypothalamic signaling networks - particularly those involving kisspeptin, neurokinin B (NKB), and GABA - driving alterations in gonadotropin-releasing hormone (GnRH) pulsatility, vasomotor symptoms (VMS), and loss of reproductive cycling. Simultaneously, chronic inflammation, oxidative stress, and mitochondrial dysfunction further accelerate both ovarian and neural aging. Estrogen receptor subtypes (ERα and ERβ) play critical and region-specific roles in mediating tissue responses to hormonal withdrawal, contributing to variability in symptom expression and therapeutic outcomes. Genetic, cultural, and environmental factors - such as diet, endocrine disruptors, and APOE genotype - further influence the trajectory and severity of menopause-related changes. Emerging treatments, including neurokinin receptor antagonists and ERβ-selective modulators, offer targeted alternatives to conventional hormone therapy. This review frames menopause not as a singular endocrine endpoint but as a neuroimmune transition, highlighting the need for mechanistic insight and personalized therapeutic approaches to improve health outcomes during reproductive aging.

## Introduction

The transition through menopause is marked by a complex interplay of systemic and central changes, driven primarily by the gradual depletion of ovarian follicles. This biological progression not only signals the end of natural fertility but also initiates a cascade of physiological adaptations that influence various tissues and regulatory networks. Among the most frequently reported and clinically disruptive consequences of this transition are vasomotor symptoms (VMS), which affect a significant proportion of menopause-bearing individuals and are often resistant to simplistic explanatory models.

This review explores the continuum from ovarian aging to neuroendocrine reprogramming, positioning VMS as downstream manifestations of these interconnected processes. Declines in estrogen production, estrogen receptor (ER) density shifts, alterations in gonadotropin neurons, coupled with changes in inflammatory signaling, collectively contribute to altered central thermoregulatory control. These changes intersect with specific hypothalamic circuits, such as those involving kisspeptin and neurokinin B, which are increasingly implicated in the emergence of hot flashes and related symptoms.

By examining the underlying biology of reproductive aging - beginning with reproductive senescence and extending through central neuroendocrine adaptation - this manuscript seeks to present an integrated framework for understanding menopause-related symptoms. In doing so, it also evaluates current and emerging therapeutic modalities, including hormonal and non-hormonal treatments, that aim to mitigate the physiological impact of these transitions. The goal is not only to chart the mechanistic landscape of menopause but also to connect it to clinically relevant strategies that improve quality of life for aging individuals.

## Methods

This narrative review aims to synthesize current knowledge on the neuroendocrine, metabolic, and immunological processes involved in reproductive aging and the menopausal transition. To capture a broad yet relevant scope of literature, a comprehensive search of peer-reviewed articles was conducted using PubMed, Scopus, and Google Scholar databases through July 2025. Search terms included combinations of keywords such as “reproductive aging,” “menopause,” “kisspeptin,” “KNDy neurons,” “estradiol withdrawal,” “vasomotor symptoms,” “neuroendocrine regulation,” “estrogen receptors,” “HPA axis,” and “inflammation.”

Priority was given to articles published within the last 15 years, although seminal and foundational studies outside this range were included when contextually necessary. Both human and relevant animal studies were considered, particularly those exploring hypothalamic signaling, estrogen receptor dynamics, and systemic inflammatory responses related to menopause.

Literature selection was guided by thematic relevance to the physiological and pathophysiological mechanisms of reproductive aging. Studies were reviewed for methodological rigor, novelty, and translational significance. The review also integrates emerging therapeutic perspectives, including non-hormonal strategies targeting neurokinin signaling and estrogen receptor subtypes.

This review does not follow a systematic review or meta-analysis protocol; rather, it provides an interpretive synthesis of the literature, highlighting both well-established mechanisms and areas of ongoing debate or limited evidence. The narrative approach allows for a flexible, integrative exploration of the multidimensional nature of menopausal biology and its broader implications for women’s health.

## Molecular changes and cellular physiology during the menopausal transition

### Neuroendocrine and ovarian dynamics across the menstrual cycle and menopausal transition

During a woman’s peak reproductive years, estrogen levels fluctuate predictably throughout the menstrual cycle, regulated primarily by follicle-stimulating hormone (FSH) and luteinizing hormone (LH). FSH stimulates ovarian follicles, which are fluid-filled sacs containing eggs, to produce estrogen. Once estrogen reaches a certain threshold, it signals the brain to suppress FSH and trigger a surge in LH, which prompts ovulation - the release of an egg from its follicle. The remaining follicle then produces progesterone and additional estrogen to prepare for potential pregnancy. As progesterone and estrogen levels rise, FSH and LH levels decline. If pregnancy does not occur, progesterone levels drop, menstruation begins, and the cycle restarts (1).

**Figure 1:**
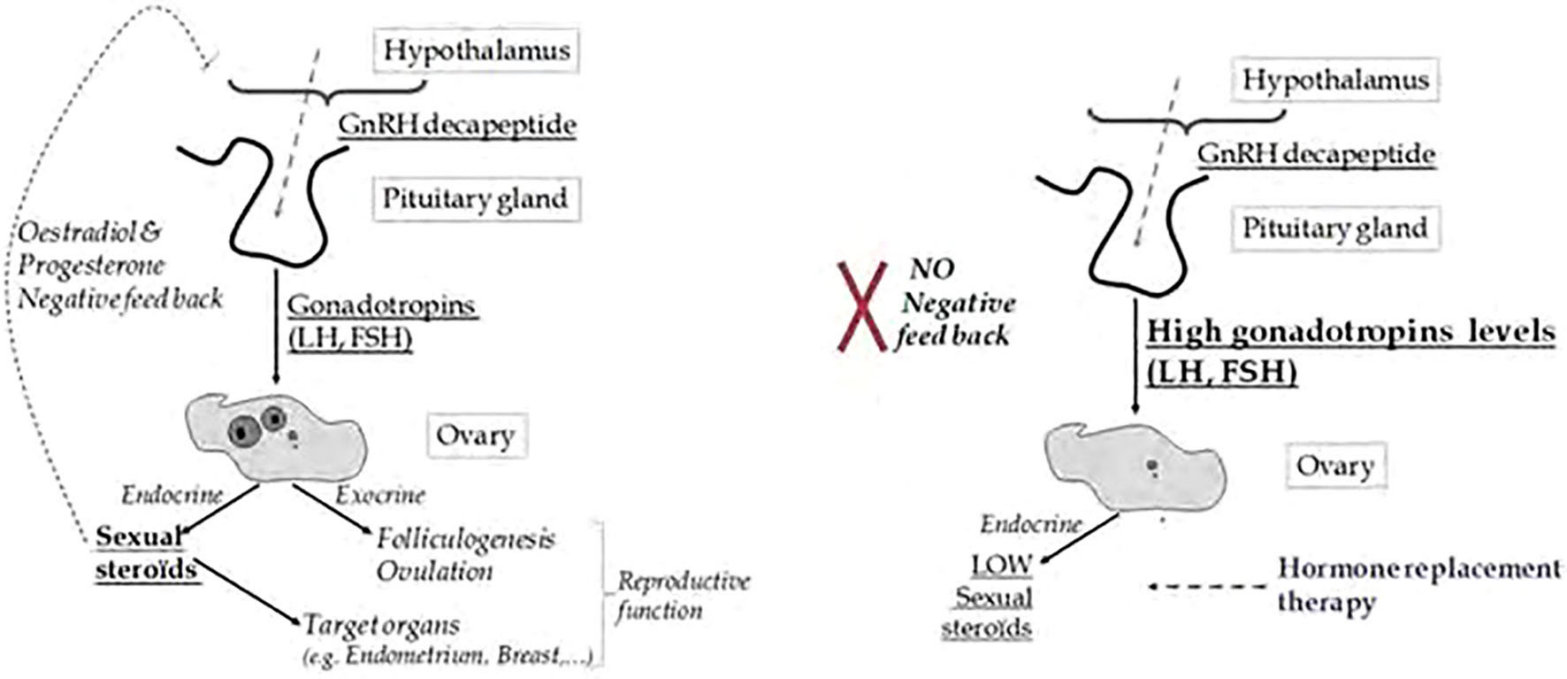
Comparative Diagram of HPG Axis Signaling in Normal vs. Menopausal Women: HPG axis signaling in normal reproductive-aged women versus menopausal women. In normal conditions, ovarian estrogen and progesterone provide negative feedback to the hypothalamus and pituitary, maintaining balanced GnRH, FSH, and LH levels, which support regular ovulation and hormone cycles. In menopause, ovarian failure leads to reduced estrogen and progesterone, disrupting feedback regulation. This results in elevated GnRH, FSH, and LH levels, reflecting the loss of ovarian responsiveness and contributing to menopausal symptoms such as vasomotor instability. (Image adapted from 3).

Menopause is characterized by the degeneration of ovarian follicles and a corresponding increase in gonadotropins, such as follicle-stimulating hormone (FSH) and luteinizing hormone (LH), due to reduced negative feedback from declining levels of ovarian steroids, particularly estrogen (2). These hormonal changes are accompanied by alterations in the hypothalamic-pituitary-gonadal (HPG) axis, with disruptions in gonadotropin-releasing hormone (GnRH) regulation contributing to the compensatory increases in FSH and LH. **Figure 1** provides a simplified overview of the hypothalamic-pituitary-gonadal (HPG) signaling pathway:

There are three known neurokinin receptor subtypes - NK1R, NK2R, and NK3R - encoded by the *Tacr1*, *Tacr2*, and *Tacr3* genes, respectively. The *Tacr3* gene, which encodes the NK3 receptor (NK3R), is located on chromosome 8 (4). Neurokinin B (NKB), the endogenous ligand for NK3R, plays a critical role in reproductive hormone regulation, particularly during the menopausal transition. In non-human primates such as rhesus monkeys, ovarian failure is associated with increased gonadotropin secretion, mirroring patterns observed in menopausal women. During periods of hypoestrogenism or after prolonged ovariectomy, NKB expression is markedly upregulated in the arcuate (infundibular) nucleus - a key hypothalamic region involved in reproductive endocrine control (5).

Kisspeptin neurons are distributed in two primary hypothalamic regions: the arcuate (infundibular) nucleus (ARC) and the rostral periventricular area of the third ventricle (RP3V). The ARC contains KNDy neurons, which co-express kisspeptin, neurokinin B, and dynorphin. These neurons serve as central modulators of gonadotropin-releasing hormone (GnRH) secretion by driving its episodic release from the median eminence into the hypophysial portal system, thereby coordinating pulsatile luteinizing hormone (LH) secretion (6). Specifically, RP3V kisspeptin neurons are crucial for mediating estradiol-induced positive feedback, which is necessary for triggering the preovulatory LH surge in females - a mechanism that is absent in males (7).

Norepinephrine (NE) also plays a significant role in the regulation of the luteinizing hormone (LH) surge. It activates neurons in the anteroventral periventricular nucleus (AVPV), a critical hypothalamic region involved in positive estrogen feedback, thereby facilitating both the initiation and maintenance of the LH surge (8, 9). NE can also stimulate GnRH neurons directly in the medial preoptic area (MPA) (9). In healthy women, plasma norepinephrine (NE) levels follow a cyclical pattern, peaking around ovulation and during the early luteal phase (10). This neuroendocrine rhythm may not only support ovulatory physiology but could also contribute to behavioral changes observed during this phase - such as increased energy levels, heightened motivation, and enhanced productivity - commonly reported by women around ovulation.

Kisspeptin serves as the principal output signal to GnRH neurons, strongly promoting GnRH and subsequent gonadotropin release across mammalian species. Central administration of full-length kisspeptin or its active fragment Kp-10 robustly enhances gonadotropin secretion in rodents, ruminants, and primates. The abolishment of this effect by GnRH antagonists confirms that kisspeptin acts upstream of GnRH (6). Supporting this, qPCR analyses have identified elevated expression of kisspeptin and its receptor in the medial basal hypothalamus of postmenopausal monkeys. Similar findings were observed in ovariectomized young rhesus monkeys, with estrogen replacement reversing these changes - highlighting the modulatory role of estrogen on hypothalamic signaling (11–14).

In humans, neuronal hypertrophy within the infundibular nucleus (the human equivalent of the arcuate nucleus) is a well-documented hallmark of menopause. These enlarged neurons co-express estrogen receptor α (ERα), NKB, substance P, and kisspeptin mRNA, alongside increased tachykinin and kisspeptin gene transcription (14, 15). Although aging men also display some hypertrophy of these neurons, the changes are markedly milder, reflecting the gradual decline in testicular function compared to the overt depletion of ovarian follicles in women (16).

Genome-wide association studies (GWAS) and meta-analyses further implicate NKB signaling in menopausal physiology. Single nucleotide polymorphisms (SNPs) in *TAC3*, the gene encoding NKB, have been linked to vasomotor symptoms such as hot flashes in postmenopausal women. Additionally, inactivating mutations in *TAC3* and *TACR3* have been associated with hypogonadotropic hypogonadism, a condition marked by absent puberty, low LH levels, and insufficient gonadal steroid production (17).

Polymorphisms affecting NKB signaling may heighten neuronal activity in the KNDy network, possibly promoting a hypergonadotropic state by amplifying GnRH secretion. While GnRH levels do increase post-menopause, they remain relatively stable during the transition phase, in contrast to the more abrupt rise in LH and FSH levels. This disparity reflects diminished pituitary sensitivity to estrogen with age, reducing negative feedback and enhancing gonadotropin output (18). Notably, these hormonal changes begin in the early menopausal transition, even before major declines in circulating estrogen or progesterone are apparent. A key shift occurs in the mid-cycle LH surge, which becomes less frequent but more prolonged - disrupting ovulation and contributing to menstrual irregularities (19).

This gradual decline in ovarian function is partly driven by reduced sensitivity of granulosa and theca cells to gonadotropins (FSH and LH), particularly in the later premenopausal years. As a result, estrogen and progesterone production becomes impaired - especially the theca cell response to the mid-cycle LH surge - contributing to irregular ovulation and menstrual dysfunction (20). These hormonal disturbances further disrupt the feedback loop between the ovaries and the hypothalamus, impairing GnRH regulation and reinforcing the neuroendocrine instability characteristic of the menopausal transition (21).

Notably, kisspeptin influences early follicle development by suppressing FSH receptor (FSHR) expression, which affects primary and secondary follicle recruitment (22). In 6- and 10-month-old rats, ovarian injection of kisspeptin led to a reduction in the number of antral follicles, including atretic ones, while administration of a kisspeptin receptor antagonist (p234) had the opposite effect. *In vitro* studies further revealed that kisspeptin counteracts the FSHR upregulation caused by isoproterenol (a beta-adrenergic agonist), functioning as a negative regulator.

Additionally, kisspeptin has been shown to increase serum levels of anti-Müllerian hormone (AMH), a dimeric glycoprotein secreted by preantral and small antral follicles. AMH plays a critical role in regulating follicle recruitment by inhibiting the activation of primordial follicles and modifying their sensitivity to follicle-stimulating hormone (FSH) (22, 23). Experimental studies indicate that local administration of kisspeptin elevates serum AMH concentrations, whereas treatment with the kisspeptin receptor antagonist p234 reduces AMH levels in both young and middle-aged rats (22). These findings suggest that kisspeptin may suppress preantral follicular development by simultaneously promoting AMH expression and downregulating FSH receptor (FSHR) expression within the ovary.

Granulosa cells within antral follicles are the primary source of AMH, secreting it into both the follicular fluid and circulation. Growing follicles continue to produce AMH until they reach the developmental stage required for dominant follicle selection in response to exogenous FSH stimulation (24). At this point, AMH gene transcription is downregulated, likely to allow enhanced FSH-FSHR interactions necessary for the final maturation of the dominant follicle. Therefore, AMH expression normally exhibits cyclical variations that correspond to stages of follicular development.

Interestingly, the decline in AMH transcription following dominant follicle selection may reflect a broader biological principle that extends to menopause. In both contexts - cyclic downregulation during ovulatory cycles and chronic decline during reproductive aging - a reduction in AMH appears to correlate with an increased drive for gonadotropin responsiveness and follicular recruitment. This suggests that diminished AMH signaling may serve as a compensatory cue aimed at maintaining folliculogenesis under conditions of reduced ovarian reserve.

Importantly, studies in AMH-null mice have shown that the absence of AMH leads to accelerated depletion of the primordial follicle pool. By 13 months of age, AMH knockout mice displayed a threefold reduction in follicle number compared to wild-type controls, with over half ceasing ovulation by 16–17 months, while most wild-type mice continued to cycle normally (24, 25). These findings reinforce the utility of AMH as a sensitive biomarker for early detection of diminished ovarian reserve and impending ovarian insufficiency (26).

### Glutamatergic and GABAergic modulation of GnRH neuron excitability

Glutamate, a crucial neurotransmitter in the central nervous system, is involved in regulating gonadotropin-releasing hormone (GnRH) secretion in the hypothalamus. Immunocytochemical studies have shown that VGluT2, a transporter that loads glutamate into vesicles, colocalizes with GnRH neurons. This colocalization is particularly evident in the preoptic area, where GnRH neuron cell bodies are located, and the median eminence, where their terminals reside in rodent brains. Interestingly, research suggests that GnRH neurons in female rats may have the ability to release glutamate independently (27, 28). In aging female rats, declining glutamate levels coincide with changes in GnRH secretion, resulting in delayed and diminished luteinizing hormone (LH) surges (29). Interestingly, estradiol suppresses glutamatergic transmission to KNDy neurons, reducing spontaneous excitatory postsynaptic currents (sEPSC) frequency (30, 31). These findings suggest that both reduced glutamate levels and decreased sensitivity of GnRH neurons to glutamate-associated stimulation contribute to age-related changes in GnRH release.

**Figure 2:**
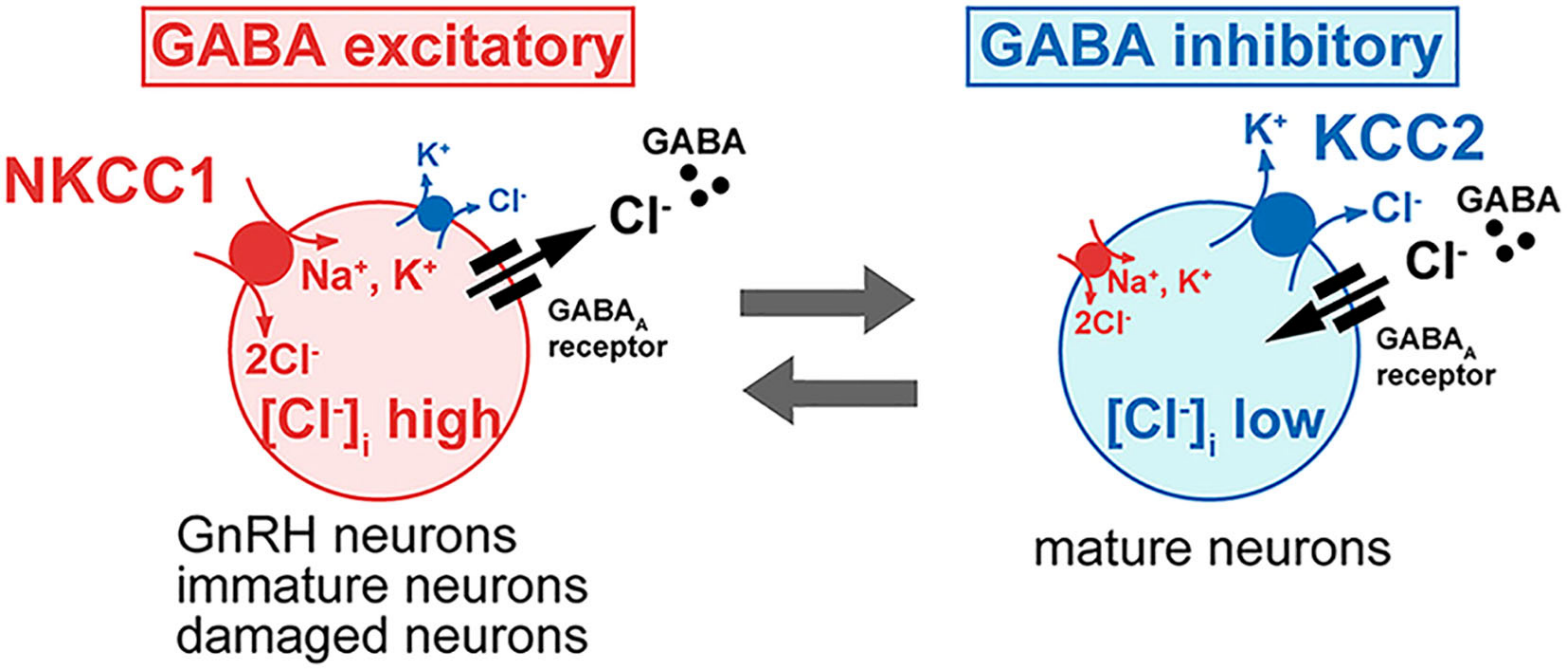
Influence of Intracellular Chloride on GABA-A Receptor Function in GnRH Neurons: This figure illustrates how intracellular chloride (Cl^-^) levels affect GABA_A_Rs receptor signaling and GnRH neuron excitability. In most neurons, low intracellular Cl^-^ levels result in GABA_A_Rs receptor activation causing Cl^-^ influx, hyperpolarization, and inhibition. In contrast, GnRH neurons exhibit elevated intracellular Cl^-^ levels, causing GABA_A_Rs receptor activation to lead to Cl^-^ efflux, membrane depolarization, and increased excitability. This shift from inhibitory to excitatory GABA signaling is critical for GnRH neuron activity and hormone release. (Image adapted from 32).

Gamma-aminobutyric acid (GABA) is another important neurotransmitter involved in the regulation of gonadotropin-releasing hormone (GnRH) neuron activity. Research on rodents has revealed that GnRH neurons receive input from GABA-containing neural pathways, suggesting that GABA plays a direct role in modulating GnRH secretion through synaptic communication (**Figure 2**). Customarily thought of as an inhibitory neurochemical, GABA is typically associated with the suppression of glutamate activity. Studies in middle-aged female rats showed increased GABA release in the medial preoptic area, linked to a delayed and diminished luteinizing hormone (LH) surge (33). Remarkably, researchers were able to restore normal LH surges in these rats by using a GABA antagonist (bicuculline) alongside a glutamate agonist (TPDC) (33).

However, endogenous gamma-aminobutyric acid (GABA) exhibits a dual role in regulating gonadotropin-releasing hormone (GnRH) neurons, functioning as both an inhibitor and a stimulator depending on the context. While broad activation of GABA​ ​receptors typically reduces neuronal activity, localized increases in GABA near the terminals of GnRH neurons *in vivo* have been associated with increased GnRH activity, as reflected by elevated luteinizing hormone (LH) levels (34). *In vitro* studies have further demonstrated this duality, showing that GABA can either suppress or enhance GnRH release or neuronal activity based on factors such as the brain region and the animal’s age (35–38). Interestingly, GABAergic inputs also appear to facilitate action potential generation in GnRH neurons (39).

Research indicates that GABA receptor subtypes play distinct roles in regulating glutamate-induced GnRH secretion. GABA_A_R ​ receptor antagonists have been shown to block glutamate-stimulated GnRH release *in vitro* without impacting basal secretion (35). Conversely, activation of GABA_A_R receptors by muscimol (MUS) stimulates basal GnRH release and, when combined with glutamate (GLU), produces an additive stimulatory effect, highlighting a cooperative relationship between these neurotransmitters. On the other hand, GABA_B_R​ receptor activation appears to exert an inhibitory influence on glutamate’s effects. Studies demonstrate that baclofen (BAC), a GABA_B_R​ receptor agonist, inhibits GLU-induced GnRH secretion, while this effect is reversed by GABA_B_R​ receptor antagonists (32).

In certain adult neurons from various brain regions, stimulation of GABA_A_R ​ receptors can lead to depolarization and even excitation sufficient to generate action potentials. A study using gramicidin-perforated patch, current-clamp experiments revealed that blocking GABA_A_Rs depolarized and/or excited *most* neurons, consistent with an inhibitory role for endogenous GABA (38). However, a notable limitation in brain slice experiments is that glutamatergic and GABAergic networks remain largely intact. When GABA_A_R antagonists are applied to a brain slice, they block GABA-mediated inhibition not only on GnRH neurons but also on other neurons in the slice. This widespread inhibition removal disrupts the balance between excitation and inhibition in the network, causing hyperactivation of glutamatergic neurons, excessive glutamate release, hyperexcitability, and seizure-like discharges (32). In other words, widespread inactivation of GABA_A_R and reduced GABAergic signaling overshadows the true effect of GABAergic stimulation on GnRH neurons, specifically.

A study by Moenter and DeFazio (39) addressed this complexity by isolating the GABA_A_R activity in GnRH neurons while blocking ionotropic glutamate receptors (iGluRs). This experimental setup increased the firing rate of GnRH neurons, demonstrating that synaptic activation of GABA_A_Rs can be excitatory under conditions when iGluR activity is blocked and contribute to action potential firing in these neurons. Blocking iGluRs minimizes broader network interactions, allowing a clearer evaluation of GABAergic effects on GnRH neurons. In contrast, when iGluRs were left intact, subsequent GABA_A_R antagonism led to a decreased firing rate in GnRH neurons. This effect could reflect either the removal of direct GABAergic inhibition on GnRH neurons. These findings indicate that endogenous activation of GABA_A_Rs play a role in driving GnRH neuron activity.

**Figure 3:**
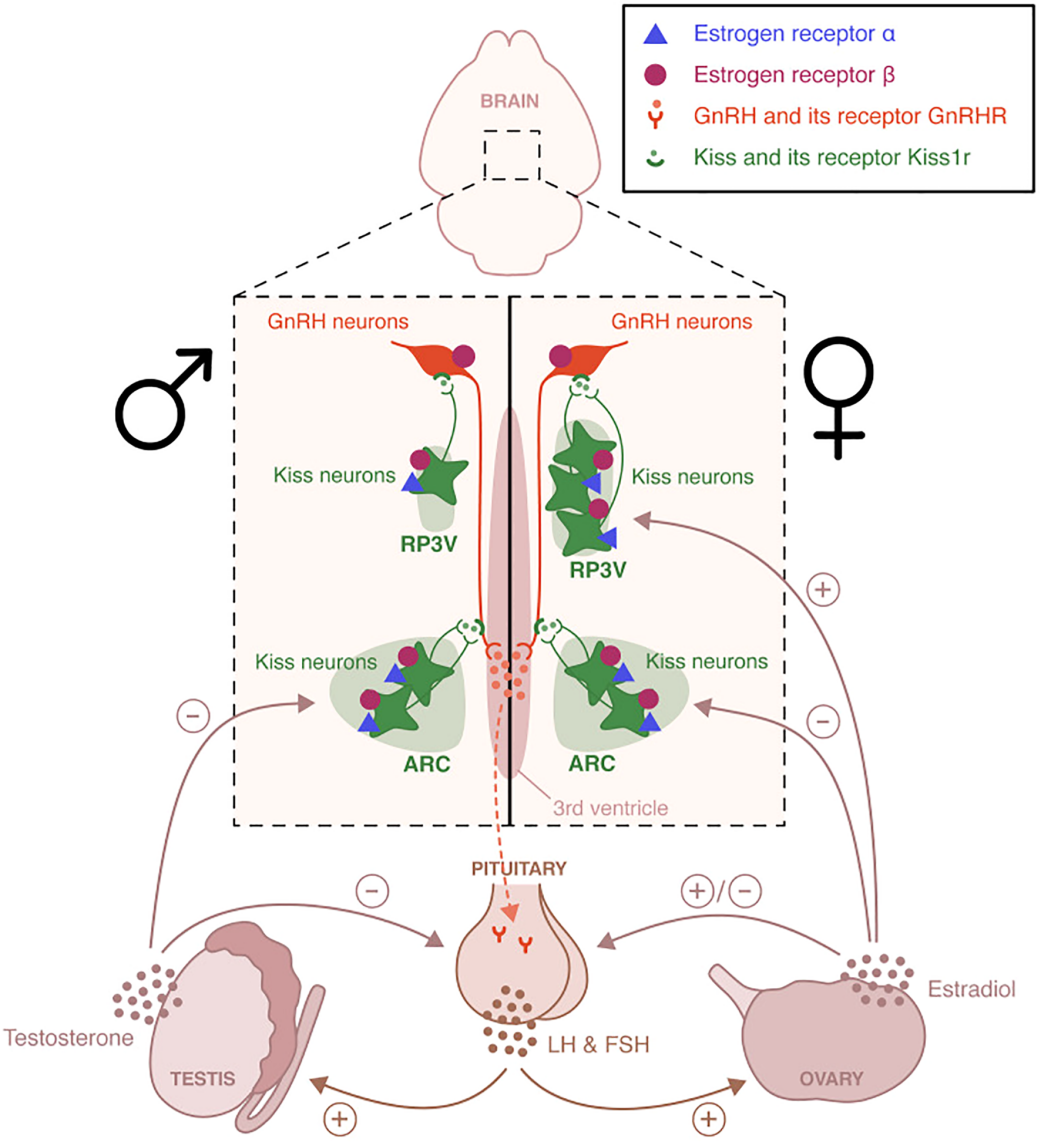
Distinct hypothalamic kisspeptin neuron populations mediate estrogen feedback regulation of GnRH secretion. Kisspeptin neurons in the arcuate nucleus (ARC) co-express neurokinin B and dynorphin (KNDy neurons) and are involved in negative feedback, regulating pulsatile GnRH and LH secretion. In contrast, kisspeptin neurons in the rostral periventricular area of the third ventricle (RP3V) are critical for mediating positive estradiol feedback and initiating the preovulatory LH surge in females. This RP3V-driven mechanism is not functional in males, reflecting sex-specific developmental differentiation. NE is not shown. Image obtained from: Torres et al. (7).

Additional factors influencing GnRH neurons polarity has to do with the relative preponderance Cl- anions within these neurons. GnRH have an unusual predilection towards elevated intracellular Cl- in adulthood, GABA activity, and a consequent excitatory bias (32, 40) **Figure 3** shows this juxtaposition.

Glutamate and GABA play complementary roles in the regulation of gonadotropin-releasing hormone (GnRH) secretion. Glutamate primarily acts as an excitatory neurotransmitter, stimulating GnRH neuron activity - often through its interaction with kisspeptin neurons - and contributing to the preovulatory luteinizing hormone (LH) surge, especially in female rodents. Conversely, GABA typically serves as an inhibitory signal, dampening neuronal activity and suppressing glutamatergic input. Notably, GABA can also exert excitatory effects on GnRH neurons depending on the receptor subtype and physiological context, highlighting its dual modulatory capacity.

### Neuroendocrine feedback dynamics: the role of estradiol, kisspeptin/KNDy neurons, GABA, and glutamate in the regulation of ovulatory cycles

To restate, estradiol’s negative feedback - primarily acting on arcuate nucleus (ARC) kisspeptin neurons - is mediated by subthreshold estrogen levels during the follicular phase. In contrast, rising estradiol levels in the late follicular phase trigger a positive feedback mechanism that suppresses ARC kisspeptin neuron activity while activating kisspeptin neurons in the anteroventral periventricular nucleus (AVPV). This shift underpins the transition from pulsatile to surge-like secretion of gonadotropin-releasing hormone (GnRH) and luteinizing hormone (LH), which is essential for ovulation. Glutamatergic transmission is a key modulator in this switch: estradiol positive feedback enhances excitatory glutamatergic input to AVPV kisspeptin neurons and decreases it in the ARC, whereas negative feedback has the opposite effect. These effects are dependent on estrogen receptor alpha (ERα) expression on kisspeptin neurons (41).

Dynorphin, a neuropeptide found within KNDy neurons, has been shown to suppress excitatory glutamatergic signaling to these same neurons, likely by acting at presynaptic terminals to inhibit transmitter release (41). During the phase of estradiol positive feedback, inhibitory GABAergic tone on AVPV kisspeptin neurons is reduced, allowing for enhanced neuronal activation (30, 42). Although estradiol is known to increase glutamatergic input to GnRH neurons during proestrus, this input is relatively sparse and occurs at a low frequency. As a result, even though glutamate can depolarize GnRH neurons, its direct effect on firing activity under physiological conditions may be modest.

Collectively, these findings suggest that estradiol’s modulation of glutamatergic signaling to kisspeptin neurons - rather than to GnRH neurons - is the more critical driver of the preovulatory GnRH surge. Moreover, kisspeptin itself may enhance GnRH neuron excitability indirectly via other afferent pathways that increase both GABAergic postsynaptic current (PSC) frequency and amplitude, as well as glutamatergic excitatory postsynaptic current (EPSC) frequency (41).

Importantly, during the early to mid-follicular phase, lower estradiol levels support negative feedback, characterized by reduced glutamatergic input to AVPV kisspeptin neurons and increased input to ARC kisspeptin neurons (41). This maintains pulsatile GnRH secretion without initiating the preovulatory surge. In the absence of sufficient estradiol-driven positive feedback, ARC kisspeptin (KNDy) neurons continue to generate GnRH pulses, but the synchronized, high-amplitude GnRH/LH surge fails to occur due to inadequate activation of AVPV kisspeptin neurons.

In addition to kisspeptin, GABA_A_R signaling in GnRH neurons serves as a key modulatory mechanism. Within the AVPV, two distinct afferent pathways converge onto GnRH neurons: a rapid, low-frequency GABAergic input and a slower-onset, high-frequency kisspeptin input. Both pathways can enhance the firing rate of GnRH neurons, but kisspeptin produces a more pronounced excitatory effect, particularly in the context of the delayed yet sustained GnRH and LH surges characteristic of positive feedback (43). Direct stimulation of GABA_A_R on GnRH neurons also promotes their activation, implying that increased GABAergic input may act synergistically with kisspeptin to trigger GnRH release (43, 44).

It has been proposed that GABAergic signaling from RP3V neurons represents the baseline mode of communication with GnRH neurons throughout most of the estrous cycle. Kisspeptin signaling appears to be selectively recruited on the day of the LH surge, possibly via distinct RP3V subpopulations (43). Consistent with this, estrogen receptor α deletion in GABAergic neurons disrupts positive feedback and abolishes the LH surge, while optogenetic activation of AVPV GABAergic neurons can elicit LH release (44). In the arcuate nucleus, the spontaneous firing rate and glutamatergic input to KNDy neurons are both elevated in Kisspeptin Estrogen Receptor Knockout (KERKO) mice, suggesting region-specific adaptations to the loss of estrogen signaling (41).

Whether increased GABAergic transmission plays a central role in the elevated gonadotropin output observed in the absence of negative feedback during menopause remains uncertain. Nonetheless, studies have linked enhanced GABAergic input from the arcuate nucleus (ARN) to GnRH neurons with increased GnRH and LH pulse frequency in individuals exhibiting disrupted estrogen-positive feedback. This pattern is commonly seen in estrogen-related disorders, where diminished progesterone levels, reduced progesterone receptor activity, and relative estrogen dominance coexist (45). Notably, in the RP3V, fast-acting GABA_A_R signaling from kisspeptin/GABA co-expressing neurons is known to stimulate GnRH neuron activity, raising the possibility that a comparable excitatory GABAergic pathway may exist within the ARC/ARN. While such a mechanism could influence reproductive hormone regulation, its specific role in the neuroendocrine shifts of menopause remains inadequately defined and merits further investigation.

As a continuation of the discussion on altered progesterone signaling, it is important to consider the specific effects of progesterone receptor (PGR) loss within kisspeptin neurons. In a study by Gal et al. (46), the *Pgr* gene was selectively deleted in mouse kisspeptin neurons to investigate the consequences for reproductive function. Despite retaining kisspeptin expression in the hypothalamus, responsiveness to GnRH at the pituitary level, and the capacity for ovulation following gonadotropin stimulation, female mice lacking PGR in kisspeptin neurons gradually lost estrous cyclicity, failed to generate an LH surge in response to rising estradiol, and ultimately became infertile. These findings indicate that while kisspeptin synthesis remains intact, PGR is essential for proper kisspeptin secretion. PGR expression in kisspeptin neurons was shown to be under the control of estrogen receptor alpha (ERα), as evidenced by the absence of PGR in the anteroventral periventricular nucleus (AVPV) of ERα-knockout mice and by the cyclical changes in hypothalamic PGR expression that mirror estradiol fluctuations throughout the estrous cycle in wild-type mice Gal et al. (46).

Further, in Kiss1-Pgr knockout mice, serum LH levels did not increase under LH surge-inducing estradiol protocols as they did in wild-type controls. This suggests that estradiol’s ability to elicit an LH surge depends on the presence and activation of PGR. However, because kisspeptin mRNA and protein levels in the AVPV of these knockout mice remained unchanged relative to controls, PGR likely does not regulate *Kiss1* gene expression directly. Instead, the evidence points toward a role for PGR in modulating kisspeptin release mechanisms. Notably, despite this disruption in positive feedback pathways, impaired PGR activity does not appear to interfere with the negative feedback regulation mediated by arcuate nucleus (ARC) kisspeptin neurons (47).

During the menopausal transition, circulating estradiol levels decline sharply, disrupting both the positive and negative feedback mechanisms that normally regulate kisspeptin neuron activity. In the absence of estradiol’s inhibitory control - primarily mediated through estrogen receptor alpha (ERα) on KNDy neurons - arcuate kisspeptin (KNDy) neurons become disinhibited. This loss of negative feedback leads to increased expression of kisspeptin and neurokinin B (NKB), reduced dynorphin-mediated inhibition, and heightened neuronal excitability and synchrony. As a result, KNDy neurons in the arcuate nucleus undergo compensatory hypertrophy and become hyperactive, driving an increase in the frequency and amplitude of pulsatile GnRH and LH secretion, despite the absence of ovulatory cycles. Meanwhile, kisspeptin neurons in the anteroventral periventricular nucleus (AVPV), along with their associated GnRH targets, lose the excitatory glutamatergic and GABAergic input typically facilitated by high estradiol levels. This renders the AVPV circuit functionally quiescent, contributing to the cessation of the ovulatory cycle and reproductive capacity.

Since kisspeptin neurons are typically inhibited by GABAergic inputs - unlike GnRH neurons - GnRH neuron firing is shaped by both direct stimulation from kisspeptin neurons (which are excited by glutamatergic input) and direct excitatory GABAergic input acting via GABA_A_R. Ablation of estrogen production reduces glutamatergic activation of AVPV kisspeptin neurons and diminishes GABAergic stimulation of GnRH neurons - both of which are critical for generating the preovulatory LH surge. In contrast, in the arcuate nucleus, estrogen loss enhances glutamatergic signaling between KNDy neurons and increases GABAergic excitation of GnRH neurons, leading to continuous GnRH firing and persistent gonadotropin pulses.

Lastly, the loss of progesterone receptor (PGR) expression and progesterone signaling within kisspeptin neurons has significant implications for reproductive function. Specifically, PGR loss impairs the ability of AVPV kisspeptin neurons to mediate the preovulatory LH surge, directly contributing to the anovulatory phenotype characteristic of the menopausal transition and further disrupting neuroendocrine regulation during reproductive aging.

### Inflammatory accelerants of ovarian and reproductive aging

Stressors, which are external factors that disrupt an organism’s balance, are well known to negatively impact reproductive functions. Activation of the hypothalamic-pituitary-adrenal (HPA) axis in response to stress triggers the release of stress hormones, such as cortisol, which inhibit reproductive processes across the hypothalamic-pituitary-gonadal (HPG) axis (48, 49). Inflammation, a common stress response resulting from immune system activation, also significantly affects reproductive health. While cortisol is typically anti-inflammatory in acute scenarios, inflammation itself can suppress reproductive functions, particularly during intense or prolonged immune responses (50–52).

Inflammatory processes are commonly modeled in experimental research using lipopolysaccharide (LPS), a bacterial endotoxin that activates the immune system. Acute LPS exposure has been shown to suppress luteinizing hormone (LH) secretion within hours, especially following high-dose or repeated administration over short periods (49, 53). Interestingly, chronic low-dose LPS exposure has been associated with elevated circulating levels of both LH and follicle-stimulating hormone (FSH) (54, 55). LPS also enhances aromatase activity, contributing to increased estradiol production (56). Over time, persistently elevated estrogen levels may reduce hypothalamic-pituitary-adrenal (HPA) axis sensitivity to estradiol, potentially exacerbating the physiological consequences of sudden fluctuations in estrogen signaling (9).

Moreover, prolonged low-dose estradiol exposure has been linked to increased production of interleukin-1β (IL-1β) and nitric oxide-related free radicals within the arcuate nucleus (57). These inflammatory mediators may disrupt hypothalamic-pituitary-ovarian communication. Notably, the repeated exposure to endogenous estradiol peaks during each estrous cycle has been identified as a key factor in the loss of LH surges and the eventual cessation of estrous cyclicity in rodents (9). One downstream effect of increased nitric oxide production is the formation of peroxynitrite, which can nitrate tyrosine hydroxylase in the medial preoptic area of the hypothalamus. This post-translational modification impairs GnRH neuron function and diminishes the capacity to generate a preovulatory LH surge (9). Collectively, these findings provide a mechanistic basis for how chronic inflammation and estradiol dysregulation may contribute to long-term reproductive dysfunction.

Elevated markers of inflammation, oxidative stress, LH, and FSH have also been observed in individuals with primary ovarian insufficiency, a condition often linked to accelerated aging (58). Similarly, menopausal women frequently display increased levels of FSH, LH, and inflammatory markers, indicating that inflammation and oxidative stress may play significant roles in the onset and progression of menopausal symptoms and their associated systemic effects (59).

Markers of inflammation are known to be resultant of both natural and surgical menopause (60, 61). Upon an analysis of a wide panel of inflammatory cytokines, 60 showed that IL-1β, IL-8, IL-8 and TNF-α serum levels are significantly higher in women with natural and surgically induced menopause compared with those in fertile women in the control group (60–62). Consistent with the findings in menopausal women, studies have demonstrated increased pro-inflammatory cytokine levels in middle-aged female rats (63).

The decline in ovarian steroid hormones during menopause is well-documented to enhance inflammatory processes, increasing susceptibility to immune-related disorders such as rheumatoid arthritis (64) and worsening the pathology of multiple sclerosis (65). Postmenopausal women are also more prone to heightened immune responses (66). Estrogen deficiency (E2 deficiency) specifically upregulates cytokines such as IL-1, IL-7, TNF, IFN-γ, and IL-6 (60, 61). This cytokine increase coincides with declining ovarian steroids during menopause, with higher levels of interleukins IL-6, sIL-6, IL-4, IL-2, and TNF observed in postmenopausal women. Importantly, estrogen replacement has been shown to attenuate pro-inflammatory cytokine expression (62, 67, 68).

Persistent low-grade inflammation is known to accelerate ovarian failure in conditions like premature ovarian insufficiency (58). However, the effects of natural aging on ovarian functional decline and their broader implications remain under investigation. Ovarian aging is a physiological process marked by the depletion in both the quantity and quality of the oocyte and follicular pool. While it is clear that ovarian function is central to reproductive aging, the extent to which the ovaries themselves drive reproductive dysfunction is not fully understood. A pivotal study conducted by Selmar Aschheim in 1964 transplanted ovaries from young female rats with regular estrous cycles into older rats that had lost cyclicity. The older rats did not regain cyclic activity after the transplant, suggesting that ovarian aging is not solely determined by the ovaries themselves (69). Supporting this idea, prior findings indicate that gonadotropin secretion begins to change as early as 27–28 years of age, with more pronounced alterations in follicle-stimulating hormone (FSH) secretion than luteinizing hormone (LH), particularly after age 40 (70, 71). These changes occur well before visible menstrual or ovulatory dysfunction. Interestingly, both bilaterally ovariectomized and postmenopausal women release less gonadotropin-releasing hormone (GnRH) compared to younger women with functional ovaries (72). This suggests that age-related ovarian decline is influenced by not only HPG axis dysfunction but also intrinsic changes within the ovary itself, particularly in ovarian follicles and oocytes.

With advancing age, the ovarian immune landscape undergoes significant remodeling, characterized by shifts in immune cell composition, subtype distribution, and activation states. Notably, there is a twofold increase in ovarian immune cell populations, particularly lymphocytes, in aged ovaries (73). Systemic inflammation and oxidative stress further compound the aging process. Chronic inflammatory disorders, such as inflammatory bowel disease (IBD), are associated with an earlier onset of menopause, suggesting that heightened systemic immune activation may accelerate ovarian decline (74).

Single-cell RNA sequencing of human ovaries has revealed that middle-aged ovaries are dominated by pyroptotic resident macrophages, which contribute to a proinflammatory local microenvironment (75). Similarly, studies in aged mice demonstrate that follicular depletion correlates with increased infiltration of CD4^+^ T cells, B cells, and macrophages - trends indicative of ovarian immune activation and chronic inflammation (76).

Extracellular vesicles (EVs) have emerged as key mediators in the propagation of pyroptotic signaling. These vesicles facilitate the intercellular transmission of inflammasome components, thereby perpetuating inflammatory cascades (77). In a rat model of natural reproductive aging, ovaries were found to release EVs containing inflammasome proteins into both the bloodstream and cerebrospinal fluid (CSF), mirroring the protein signature observed in the CSF of peri-menopausal women (78). Strikingly, when introduced into young female rats, EVs derived from peri-menopausal human serum were capable of inducing inflammasome activation within the brain, indicating that these vesicles can cross the blood-brain barrier and elicit central immune responses. EVs containing inflammasome elements have also been implicated in neuroinflammation following traumatic brain injury and stroke (79, 80). Consistent with these findings, elevated levels of interleukin-1β (IL-1β) have been detected in both the serum and brains of reproductively senescent female rats, as compared to younger females and age-matched males (78).

Cellular senescence is a state of irreversible cell-cycle arrest triggered by various stressors and characterized by distinct changes in morphology, metabolism, gene expression, and epigenetic landscape (81). During ovarian aging, particularly in murine models, senescent cells accumulate within the ovarian stroma. These cells secrete a complex mixture of pro-inflammatory cytokines, chemokines, growth factors, and proteolytic enzymes, collectively referred to as the senescence-associated secretory phenotype (SASP). The SASP promotes local and systemic inflammation and reinforces the senescent state through both paracrine and autocrine signaling mechanisms (82).

Senescent ovarian cells often accumulate undegraded proteins and lipids, forming intracellular aggregates known as aggresomes, which further impair cellular function. Experimental models using D-galactose have shown that exposure to this molecule accelerates ovarian senescence, resulting in increased follicular depletion and dysregulated sex hormone production (83). These findings underscore the role of cellular senescence not only as a hallmark of ovarian aging but also as a contributor to the broader endocrine and inflammatory disturbances observed during the menopausal transition.

Ovarian aging is regulated by a multifaceted interplay of biological processes, including telomere attrition, oxidative stress, mitochondrial dysfunction, disrupted protein homeostasis, and impaired autophagy. Key telomerase subunits (*TERC* and *TERT*) and telomere-associated proteins such as *TRF1/2* and *POT1A* have been shown to decline with age in both ovarian tissue and follicles (84, 85). Telomere shortening and reduced telomerase activity - particularly in granulosa cells - have been implicated in conditions like occult ovarian insufficiency, even in younger women (86). In normative aging, follicular function shows a gradual decline that accelerates around the mid-to-late 30s (87).

Estrogen deficiency during menopause has been shown to suppress telomerase activity, contributing to accelerated telomere shortening and impaired proliferation of granulosa cells - factors that ultimately hinder proper follicular development. Estrogen supplementation, by contrast, has demonstrated the ability to restore telomere length, underscoring its role in preserving cellular longevity and reproductive potential (88). Resveratrol, a polyphenolic compound, has also garnered attention for its potential to delay age-related reproductive decline. In murine models, prolonged oral administration of resveratrol has been associated with increased telomerase activity and delayed fertility deterioration (89, 90). Additionally, preclinical and early clinical studies suggest that resveratrol pretreatment may enhance both the quantity and quality of oocytes (91), although these benefits appear diminished in women over 35 years of age (89). Despite these promising cellular effects, resveratrol has not consistently improved clinical pregnancy outcomes and, in some cases, may be detrimental (89, 91). Evidence points to a biphasic effect, wherein resveratrol may impair endometrial decidualization—potentially compromising implantation - even when high-quality embryos are transferred (92, 93). However, limiting resveratrol administration to the proliferative phase of the menstrual cycle, rather than the decidual phase, may allow for ovarian benefits without causing further complications (94).

Mitochondrial aging significantly impacts oocyte quality by impairing ATP production and disrupting spindle assembly during meiosis, thereby increasing the risk of chromosomal segregation errors. Proinflammatory cytokines, such as interleukin-6 (IL-6), have been shown to impair microtubule organization and chromosomal alignment in mouse oocytes, resulting in meiotic spindle defects (95). Furthermore, knockdown of *Il1a* in aged mice has been associated with improved pregnancy rates and increased litter sizes, underscoring the deleterious role of inflammatory cytokines in reproductive aging (96).

Environmental factors such as radiation, chemotherapeutic agents, and toxins also contribute to ovarian aging by inducing DNA damage, including single- and double-strand breaks (97, 98). Deficiencies in DNA repair mechanisms - exemplified by BRCA1 mutations - are associated with diminished ovarian reserve and compromised oocyte quality in both humans and animal models (99, 100).

Oxidative stress, primarily driven by reactive oxygen species (ROS) such as superoxide and hydrogen peroxide, further exacerbates mitochondrial dysfunction. The resulting oxidative damage contributes to spindle disassembly and aneuploidy - key features of age-related reproductive decline (101). Promisingly, supplementation with nicotinamide riboside, a precursor of NAD^+^, has been shown to restore mitochondrial function, reduce oxidative damage, and improve ovarian performance in aged models (102).

With advancing age, the ovarian free radical/antioxidant balance undergoes significant alterations, characterized by diminished antioxidant defenses and increased oxidative damage. This age-associated oxidative stress compromises oocyte quality and depletes ovarian reserve, primarily by inducing granulosa cell (GC) apoptosis and impairing GC–oocyte communication (103). For example, germinal vesicles (GV) and metaphase I (MI) stage oocytes from older mice exhibit elevated levels of ROS (104). In non-human primates, oxidative damage in ovarian GCs intensifies with age and correlates with downregulation of genes involved in oxidoreductase activity (105).

Excessive ROS disrupts redox homeostasis by inhibiting the Kelch-like ECH-associated protein 1–nuclear factor erythroid 2–related factor 2 (KEAP1–NRF2) antioxidant signaling pathway. This inhibition leads to decreased expression of cytoprotective genes and activation of forkhead box O1 (FOXO1)-mediated pro-apoptotic signaling, ultimately resulting in granulosa cell (GC) apoptosis and follicular atresia. Notably, NRF2-deficient mice are more susceptible to D-galactose-induced ovarian dysfunction and exhibit significantly impaired antioxidant capacity (106). Pharmacological activation of NRF2 using agents such as daphnetin and dimethyl fumarate has shown potential to mitigate oxidative stress-induced declines in ovarian reserve (106). Moreover, oxidative stress is a known activator of the NOD-like receptor family pyrin domain containing 3 (NLRP3) inflammasome, promoting the release of proinflammatory cytokines such as interleukin (IL)-1β and IL-18. Concurrently, ROS-mediated activation of the nuclear factor kappa-light-chain-enhancer of activated B cells (NF-κB) pathway amplifies inflammatory responses, further exacerbating ovarian dysfunction (90).

Importantly, while ROS are often associated with cellular damage, they also play essential physiological roles in the ovary. For instance, controlled ROS production is critical for ovulation, facilitating follicular rupture and oocyte release (1). However, when ROS levels exceed physiological thresholds, they contribute to meiotic errors (107), GC apoptosis (108), and increased follicular atresia (109–113).

Advanced glycation end-products (AGEs) are harmful compounds formed through non-enzymatic reactions between sugars and proteins or lipids - a process accelerated by high-temperature cooking, known as the Maillard reaction. These molecules accumulate in tissues with age and are increasingly recognized as key contributors to oxidative stress and inflammation in the ovarian environment. Within the ovary, AGEs disrupt normal function by two main mechanisms: they can bind directly to extracellular matrix components, causing structural disorganization, or activate their cellular receptor, RAGE, which initiates pro-inflammatory cascades and amplifies oxidative damage (114). This AGE–RAGE interaction has been shown to negatively affect ovarian blood supply, compromise granulosa cell function, and impair follicular development. As a result, AGEs can reduce the number of retrieved and mature oocytes, lower embryo quality, and interfere with steroid hormone production, ultimately contributing to reduced fertility (114, 115).

Interestingly, the body also produces a soluble form of the RAGE receptor (sRAGE), which circulates in fluids like blood and follicular fluid. Unlike membrane-bound RAGE, sRAGE acts as a decoy - binding AGEs before they can interact with cellular receptors and trigger inflammation. Higher levels of sRAGE have been associated with more favorable ovarian function, suggesting a protective role in counterbalancing the negative effects of AGEs (116). AGE accumulation has also been implicated in a variety of age-related and inflammatory conditions, including diabetes, cardiovascular disease, neurodegeneration, and premature ovarian failure. Notably, AGE formation is not only influenced by age, but also by diet, metabolic health, and oxidative stress levels - factors that collectively shape the ovarian aging trajectory (114).

Multiple mechanisms have been identified to explain how mitochondrial dysfunction contributes to ovarian aging, including mutations in mitochondrial DNA (mtDNA), disruptions in mitochondrial dynamics, reduced mitophagy, and impaired mitochondrial biogenesis (117). These alterations collectively lead to the buildup of defective mitochondria, increased production of reactive oxygen species (ROS), and changes in mitochondrial membrane permeability - all of which can trigger inflammatory signaling and programmed cell death.

One of the key protective mechanisms in mitochondrial quality control is mitophagy, a selective process by which damaged mitochondria and inflammasome components such as the NLRP3 complex are degraded. When functioning properly, mitophagy prevents the release of pro-inflammatory mtDNA and helps maintain cellular homeostasis (118). However, when mitophagy is compromised, dysfunctional mitochondria accumulate and can activate the NLRP3 inflammasome through excessive mitochondrial ROS, membrane instability, and leakage of mtDNA (119–121).

Inflammasome activation is further facilitated by steps such as NLRP3 deubiquitination and linear ubiquitination of the ASC adaptor protein, which promote complex assembly and cytokine maturation (122, 123). Experimental models have demonstrated that deletion of the inflammasome adaptor ASC leads to a higher number of primordial follicles and corpora lutea with age, suggesting improved ovarian preservation (76, 96). Similarly, *Nlrp3* knockout mice show enhanced ovarian reserve, more stable hormone profiles, and improved fertility during aging (124).

While mitochondria function as central signaling platforms for inflammatory responses, mitophagy - the selective degradation of damaged mitochondria - acts as a critical protective mechanism to maintain immune homeostasis (125–127). However, with advancing ovarian age, the capacity for mitophagy in oocytes diminishes, particularly after development is complete. This decline increases the likelihood of retaining and transmitting dysfunctional mitochondria to offspring (128). Mitochondrial DNA (mtDNA), which resides near the respiratory chain and lacks the protective histone packaging present in nuclear DNA, is highly susceptible to oxidative damage. As a result, its mutation rate is significantly higher than that of nuclear DNA. Studies have demonstrated that mtDNA point mutations accumulate in human oocytes with increasing maternal age and may be passed on to progeny (129). These maternally inherited mtDNA mutations can impair fertility in the next generation; however, introducing wild-type mtDNA has been shown to restore fecundity in affected offspring (130). Clinical findings further support these observations. Women carrying inherited mtDNA mutations have been reported to exhibit features consistent with premature ovarian insufficiency (POI), including reduced antral follicle counts, elevated follicle-stimulating hormone (FSH) levels, and lower anti-Müllerian hormone (AMH) concentrations - biomarkers indicative of diminished ovarian reserve (90, 131).

Another hallmark of ovarian aging is a decline in mitochondrial DNA (mtDNA) content. During oocyte maturation, particularly from the germinal vesicle (GV) stage to the fully mature oocyte, there is typically a dramatic increase in mitochondrial biogenesis to meet the high energy demands of development. However, with age, this biogenetic capacity becomes impaired, contributing to the observed reduction in mtDNA copy number (132). One of the key regulators of mitochondrial biogenesis, PGC-1α, is expressed at significantly lower levels in the cumulus cells of women with diminished ovarian reserve, highlighting a potential mechanistic link between mitochondrial decline and reduced fertility (132).

Consistent with these findings, Jin et al. (87) observed a reduction in granulosa cell proportions in aged ovaries, in line with earlier studies reporting decreased proliferation and increased apoptosis in these cells (133). This decline in granulosa cell population appears to be conserved across both human and murine models of ovarian aging (73). Transcriptomic analyses of human granulosa cells have revealed reduced expression of genes involved in mitochondrial ATP synthesis (134), while other studies have demonstrated increased oxidative stress within aged granulosa cells in both non-human primates and humans (105).

Importantly, impaired mitochondrial function also compromises steroid hormone synthesis, a critical aspect of reproductive health. Mitochondrial dysfunction limits ATP production and disrupts mitochondrial membrane potential (ΔΨm), both of which are essential for the active translocation of cholesterol into the inner mitochondrial membrane - an early and rate-limiting step in steroidogenesis. As such, disturbances in mitochondrial energetics not only reduce oocyte quality but also directly impair the ovarian endocrine function necessary for successful reproduction.

The accumulation of misfolded or damaged proteins compromises cellular viability and promotes apoptosis, particularly within ovarian tissues (41, 135). Autophagy - a key quality control mechanism responsible for degrading defective cellular components - is essential for maintaining oocyte integrity. However, autophagic efficiency declines with age, partly due to reduced expression of autophagy-related genes such as *Atg5*, *Atg7*, *ATG12*, and *Beclin1* (90, 136). This decline may also disrupt regulatory signaling pathways, including mTOR and AMP-activated protein kinase (AMPK), potentially influenced by non-coding RNAs that modulate gene expression (87). Indeed, the upregulation of specific microRNAs has been associated with impaired hormone synthesis and advanced ovarian aging (90).

Functional studies further support autophagy’s importance in reproductive aging: knockdown of *Atg7* in germ cells reduces primordial follicle counts, while *Atg5* suppression in granulosa cells leads to decreased proliferation and impaired oocyte maturation and fertilization. Evidence of diminished autophagic activity has also been observed in human granulosa cells from women of advanced maternal age (90). Encouragingly, pharmacological activation of autophagy - for example, with rapamycin - has been shown to improve oocyte quality and restore ovarian function in aged animal models (137).

Epigenetic regulation plays a key role in ovarian aging, with DNA methylation patterns undergoing notable changes over time. A gradual decline in global DNA methylation has been observed in mouse ovaries throughout the lifespan, reflecting a broader epigenetic trend seen in aging tissues. This decline may be attributed to reduced expression of DNA methyltransferases, which are responsible for maintaining methylation patterns (138). Additionally, enzymes involved in active DNA demethylation - specifically the ten-eleven translocation (TET) family proteins - also appear to be downregulated with age (139).

Loss of function in these enzymes can have direct consequences for reproductive health. For example, deletion of *Tet1* in mice has been associated with reduced fertility and an accelerated decline in reproductive capacity (140). In contrast, increasing *TET1* expression in aging human ovarian cells has been shown to enhance cell proliferation and limit apoptosis, suggesting a protective effect (139). Similarly, *Tet2* knockout models display impaired oocyte maturation and disrupted early embryonic development (105), further supporting the role of TET enzymes in maintaining reproductive competence during aging.

Advancing maternal age, particularly beyond 35–37 years, significantly increases the risk of aneuploidy (87). This heightened risk is primarily attributed to the age-related degradation of cohesion proteins that are essential for maintaining chromosomal alignment during meiosis. As these proteins deteriorate, premature chromatid separation and chromosome missegregation become more likely (101). Furthermore, human oocytes lack centrosomes and instead rely on chromatin-mediated spindle formation - a mechanism that becomes increasingly error-prone with age. The spindle assembly checkpoint (SAC), which ensures accurate attachment of chromosomes to the spindle apparatus, also declines in effectiveness in older oocytes, allowing uncorrected kinetochore errors and resulting in chromosomal instability (141, 142). Collectively, these age-related impairments lead to reduced fertilization rates, increased risk of miscarriage, and a higher incidence of embryonic developmental abnormalities (143).

Alterations in histone modification dynamics have also been implicated in the mechanisms underlying ovarian aging. In oocytes from aged females, specific histone residues - such as H4K12 - show decreased acetylation during the germinal vesicle (GV) stage and fail to undergo proper deacetylation by the metaphase II (MII) stage. This imbalance in histone acetylation is closely linked to chromosomal misalignment, a hallmark of oocyte dysfunction in advanced maternal age (144). The regulation of histone acetylation is largely governed by histone deacetylases (HDACs), enzymes that remove acetyl groups to maintain chromatin structure and genomic integrity. Notably, HDAC3 levels are markedly reduced in the oocytes of aged mice. This reduction disrupts spindle formation and chromosome alignment, contributing to increased rates of aneuploidy. Interestingly, reintroducing HDAC3 can partially rescue these defects, suggesting a potential therapeutic avenue for age-related chromosomal instability in oocytes (144).

Aging-related decline in ovarian function has been linked to decreased expression of several members of the sirtuin (SIRT) family of NAD^+^-dependent deacetylases - particularly SIRT1, SIRT3, and SIRT6. Studies in aged mouse ovaries have consistently shown reduced levels of these enzymes (145, 146). In particular, targeted knockdown of *Sirt1* in mouse oocytes leads to reduced oocyte quality and accelerated reproductive aging (145). Conversely, *Sirt1* overexpression extends ovarian lifespan, at least in part, by activating FOXO1A and suppressing mTOR signaling (147). Similarly, *Sirt6* plays a pivotal role in maintaining genomic integrity. Deletion of *Sirt6* in oocytes induces telomere dysfunction and triggers early embryonic apoptosis, while its overexpression in aged oocytes improves telomere length and lowers apoptosis rates (148). These findings suggest that sirtuins function as key modulators of oocyte quality during aging through their effects on cellular stress response, metabolic signaling, and chromatin maintenance.

If the proposed mechanisms underlying ovarian aging - such as mitochondrial dysfunction, increased oxidative stress, disrupted autophagy, and epigenetic alterations - indeed contribute to the progressive decline in reproductive capacity, then interventions that mitigate these cellular and molecular changes may hold promise for preserving ovarian function and improving reproductive outcomes. In light of this, both pharmacological and non-pharmacological strategies have garnered attention for their potential to counteract age-associated deterioration of the ovarian environment.

Pharmacological SIRT1 activators such as resveratrol and SRT1720 - both known mimetics of caloric restriction - have demonstrated protective effects on ovarian reserve (147). Resveratrol-treated mice exhibit ovarian phenotypes comparable to those observed under caloric restriction, including delayed follicular depletion and enhanced oocyte quality. However, the timing of administration appears to be critical for reproductive success. When resveratrol is given during the early, proinflammatory decidual phase - coinciding with the implantation window - it can impair decidual transformation of the endometrium and negatively impact implantation (89, 94). Restricting resveratrol use to the proliferative phase may allow it to support ovarian function without disrupting (94). To further illustrate the benefits of proper SIRT1 modulation, mice with genetic Sirt1 overexpression exhibit an extended ovarian lifespan. This is attributed to downstream activation of FOXO1A and suppression of mTOR signaling - two pathways involved in cellular longevity and reproductive aging (147, 149).

A growing body of evidence supports the role of antioxidants in mitigating ovarian aging. Compounds such as coenzyme Q10 (CoQ10), melatonin, N-acetylcysteine (NAC), and other naturally occurring antioxidants have been explored for their potential to preserve ovarian function. CoQ10, in particular, has demonstrated robust antioxidant properties that enhance mitochondrial function and reduce oxidative stress within ovarian tissue (150). In a mouse model of chemically induced ovarian failure, CoQ10 not only lowered ROS levels but also stimulated the differentiation of ovarian surface epithelium (OSE)-derived stem cells, leading to improved oocyte quality and ovarian function (151).

Further supporting these findings, Harsini et al. (152) reported that CoQ10 treatment significantly increased primordial follicle diameter, survival rates, antrum formation, and the number of mature (metaphase II) oocytes. They also observed elevated expression of the mitochondrial transcription factor Tfam in both granulosa cells and oocytes, along with higher mtDNA copy numbers - indicative of enhanced mitochondrial biogenesis.

Clinically, CoQ10 supplementation at a dose of 600 mg daily for 60 days has been shown to improve ovarian response to stimulation and enhance oocyte and embryo development in younger women (<35 years) with diminished ovarian reserve (153). However, while trends toward improved pregnancy and live birth rates were observed, they did not reach statistical significance - suggesting that combining CoQ10 with other therapeutic strategies may yield more pronounced benefits (150).

Spermidine is a naturally occurring polyamine that mimics the effects of caloric restriction and fasting by stimulating key longevity-associated pathways. It promotes autophagy, mitophagy, and mitochondrial biogenesis while facilitating the clearance of damage-associated molecular patterns (DAMPs) and reducing their accumulation (154). In aged mice, spermidine supplementation has been shown to improve oocyte quality and enhance fertility by promoting mitophagic activity (155). However, it’s important to note that excessively high doses may have the opposite effect, potentially impairing oocyte quality.

Similarly, intermittent fasting (IF) has emerged as a promising, non-pharmacological strategy to improve reproductive outcomes in the context of aging (156). IF has been shown to increase the number of antral follicles and ovulations, while also enhancing oocyte meiotic competence and early embryonic development. These improvements are attributed to better nuclear and cytoplasmic maturation in aged oocytes, as well as reductions in spindle abnormalities and chromosomal misalignment (156).

### Changes in estrogen receptor physiology during reproductive aging

Amid the myriad of feedback mechanisms that govern the hypothalamic-pituitary-gonadal (HPG) axis, estrogen receptor alpha (ERα) signaling within the arcuate nucleus (ARN), particularly in KNDy neurons, appears to play a central role in sustaining reproductive function and mediating estradiol-dependent negative feedback (157). In mouse models lacking sufficient ERα activity in this region, disruptions in GnRH pulsatility emerge, characterized by high-frequency, low-amplitude luteinizing hormone (LH) pulses - an endocrine profile resembling that of gonadectomized animals (158). Recent findings by Faure et al. (159) further support the importance of ERα, demonstrating that in the absence of progesterone, both chronic deficiency in ERα signaling and acute exposure to estetrol (E4) impair estradiol’s ability to activate kisspeptin and GnRH neurons - two neuronal populations essential for generating the LH surge. Additionally, ERα expression in the pituitary contributes to the estradiol feedback loop, with its deletion resulting in infertility in female mice (160, 161).

Estrogen receptors alpha (ERα) and beta (ERβ) are differentially expressed across peripheral and central tissues, playing distinct roles in modulating physiological responses to estrogen throughout the menopausal transition. In the vagina, both receptor types are present, but they exhibit divergent localization and age-related decline. ERα is broadly distributed in the vaginal epithelium, connective tissue, and smooth muscle, and while its levels decrease with age, they remain relatively stable compared to ERβ. ERβ is more restricted to the epithelial lining and vascular endothelium and undergoes a sharper postmenopausal reduction (162). This decline appears to be considerably reversible during perimenopause but becomes less responsive to estrogen replacement in later stages, suggesting that estrogen withdrawal initially drives the loss, while progressive tissue aging eventually dominates (163, 164).

These differential expression patterns are clinically meaningful. ERβ plays a central role in maintaining vaginal epithelial barrier integrity and vascular tone. Its heightened sensitivity to estrogen loss may account for symptoms such as vaginal dryness and atrophic changes that often persist despite hormone therapy, particularly when treatment begins after the perimenopausal window. In contrast, ERα, being more structurally embedded and hormonally stable, supports continued - though reduced - tissue remodeling capacity (162, 163). Understanding these receptor dynamics helps explain the superior efficacy of early-initiated hormone therapy in preserving vaginal tissue health.

Brain imaging studies using 16α-^18F-fluoro-17β-estradiol (^18F-FES) PET confirm that ERα is particularly enriched in the hypothalamus and pituitary - regions crucial for thermoregulation and neuroendocrine feedback. Oophorectomy in rats results in significant upregulation of ERα in these brain regions, a response attenuated by preemptive estradiol administration, indicating estrogen levels directly modulate central ER expression in a region-specific manner (165). This likely represents a neuroendocrine compensatory mechanism in response to estrogen decline and may underpin central symptoms such as vasomotor instability and mood dysregulation during menopause.

The hypothalamic regions anteroventral periventricular nucleus (AVPV), arcuate nucleus (ARC), paraventricular nucleus (PVN), and supraoptic nucleus (SON) all express estrogen receptors. ERβ is densely expressed in the PVN and SON, which contain arginine vasopressin (AVP), oxytocin (OXT), and corticotropin-releasing factor (CRF) neurons. Estrogen regulates reproductive function via kisspeptin neurons that control gonadotropin-releasing hormone (GnRH) secretion. In the female AVPV, most kisspeptin neurons co-express ERα and about 70% co-express ERβ, while in the ARC nearly all kisspeptin neurons co-express ERα but fewer than 30% express ERβ (166). Estrogen upregulates kisspeptin expression in the AVPV but downregulates it in the ARC. Mice lacking ERα show impaired estrogen responsiveness in both regions, underscoring ERα’s critical role. However, AVPV-specific ERβ knockdown disrupts estrous cyclicity and reduces fertility, indicating a contributory role for ERβ in AVPV function. Variability in reported ERβ co-expression levels may result from differences in hormonal status or detection techniques. In a rodent model of periestropause, estradiol levels remained within the normal range, yet significant neuroendocrine changes were observed: reduced progesterone levels, diminished progesterone receptor expression, and notably, decreased ERβ mRNA in the dorsal raphe nuclei (DRN) (164). These changes were associated with a reduction in tryptophan hydroxylase (TPH)-positive serotonergic neurons and lower serotonin concentrations in the amygdala and hippocampus - regions critical for mood and thermoregulation. Estradiol treatment reversed these deficits by restoring ERβ expression and enhancing serotonergic tone, particularly in the dorsal hippocampus. This suggests that the efficacy of estrogen in alleviating perimenopausal symptoms may depend less on circulating hormone levels and more on receptor expression within key brain regions.

ERβ is also central to thermoregulation, particularly in the hypothalamus where it modulates the activity of warm-sensitive neurons in the ventromedial preoptic area. Recent preclinical work using senktide - a neurokinin-3 receptor agonist that simulates hot flash physiology by activating KNDy neurons - demonstrated that ovariectomized and intact mice exhibited elevated tail temperatures mimicking VMS. Importantly, treatment with EGX358, a selective ERβ agonist, reduced these symptoms in mice carrying the APOE3 genotype. Of note, EGX358 did not mollify these symptoms in APOE3/4 heterozygotes (167). This indicates that even one copy of the APOE4 allele may blunt the VMS-relieving effects of ERβ agonism. Thus, despite the ARC KNDy neurons heavy representation of ERα (166), modulative correction of diminished ERβ activity shows favorable outcomes in perturbations of temperature regulation mechanisms within the hypothalamus.

Clinical studies corroborate these genotype-dependent effects. Phytoestrogen-based selective estrogen receptor modulators (phytoSERMs) - including compounds like genistein, daidzein, and S-equol - have demonstrated efficacy in reducing hot flashes primarily in APOE3 homozygotes, with diminished or absent effects in APOE4 carriers (105). These findings underscore the importance of incorporating APOE genotype into therapeutic decision-making and point to a key role for ERβ signaling in the modulation of vasomotor symptoms - an effect potentially disrupted by APOE4-associated alterations in neuronal or vascular estrogen responsiveness.

Interestingly, one exception to the general decline in ERβ activity during the menopausal transition is observed in adipose tissue. In postmenopausal women, ERβ expression is upregulated in adipocytes and is accompanied by increased activity of 11β-hydroxysteroid dehydrogenase type 1 (11βHSD1), an enzyme that locally converts inactive cortisone to active cortisol within fat tissue. This localized glucocorticoid reactivation contributes to hallmark features of the postmenopausal metabolic profile, including central adiposity and insulin resistance (129, 168). The increase in 11βHSD1 activity appears to be driven by residual estrogen signaling within visceral fat depots, which promotes both ERβ expression and cortisol regeneration, thereby exacerbating visceral fat accumulation and metabolic dysfunction (129).

Altogether, this tissue- and genotype-specific estrogen receptor regulation provides a unifying framework for understanding the diverse and sometimes inconsistent outcomes of menopausal hormone therapy. It explains why early intervention can optimize receptor-mediated responses, why ERβ-specific treatments may offer targeted relief for symptoms like hot flashes and urogenital atrophy, and why APOE4 carriers may require alternative or adjunctive strategies. Estrogen’s systemic effects are neither uniform nor fully reversible, and their modulation depends heavily on receptor profile, tissue type, timing of intervention, and underlying genetic context.

### Estrogen receptor subtype-specific regulation of inflammation and neural integrity consequent reproductive aging

Consequent tissue-specific changes in ERα and ERβ expression preempt many of the physiological consequences associated with the menopausal transition. This endocrine shift is well established to coincide with a rise in chronic low-grade inflammation (59), which in turn has been shown to accelerate ovarian failure (169). It is clear that elevated estrogen levels - such as those occurring during pregnancy or through pharmacological intervention - are typically associated with anti-inflammatory effects, including the suppression of several inflammatory pathways (170–172).

It has been shown that increases in estrogen lead to the suppression of many proinflammatory cytokines via inhibition of NF-κB signaling (170). Quantification of cytokine production during time-lapse microscopy revealed that 17β-estradiol suppresses IL-1β and enhances expression of interleukin-10 (IL-10), a key anti-inflammatory cytokine, during acute lipopolysaccharide (LPS) exposure (173). Notably, GPER1 activation has also been shown to rapidly downregulate TLR4 expression in macrophages, pointing to an additional estrogen receptor-mediated mechanism of dampening bacterial-induced inflammation (174).

However, estrogen also exerts immunostimulatory effects in certain contexts. For instance, it has been shown to significantly enhance pro-inflammatory responses in Kupffer cells following exposure to lipopolysaccharide (LPS) (175). Additionally, treatment with 17β-estradiol (E2) has been reported to enhance the production of interferon-gamma (IFN-γ), a type II interferon, particularly in tissues with a Th1-biased expression profile (176). Unlike type I interferons, type II IFNs are primarily produced by natural killer (NK) cells and macrophages in response to cytokines such as type I IFNs, IL-12, IL-15, and IL-18 (177). Estrogens have also been shown to enhance TLR4 expression in some settings, suggesting that their regulatory effects on this pathway depend on the relative balance of estrogen receptor subtypes expressed in specific tissues and cell types (170).

In women with autoimmune myasthenia gravis (MG), estrogen may contribute to pathological changes in the thymus under inflammatory conditions. For instance, it has been shown to promote the formation of ectopic germinal centers (eGCs) in thymic tissue - a hallmark of MG-related inflammation (178). One potential mechanism involves estrogen-induced stimulation of type I interferon (IFN-I) expression in thymic epithelial cells (TECs), which may subsequently upregulate chemokine expression. Even under resting conditions, estrogen induces low-level expression of α-acetylcholine receptor (α-AChR) and HLA-DR by TECs, potentially impairing central tolerance and increasing susceptibility to MG in women. However, once MG is established, the prevailing pro-inflammatory environment may blunt estrogen’s immunomodulatory effects. In this context, continued estrogen-driven IFN-I production may further exacerbate disease progression. Additionally, estrogens have been reported to induce type II interferon (IFN-γ) production in various cell types (176; 178), a response that - while protective against fungal and parasitic infections - can lead to excessive and maladaptive immune activity in autoimmune diseases such as systemic lupus erythematosus (SLE; 179).

It is plausible that in the presence of a robust inflammatory milieu - particularly one characterized by cellular conditions favorable to inflammation and altered estrogen responsiveness, such as shifts in estrogen receptor density, TH1 bias (in the absence of supraphysiological estrogen levels), and altered HLA-DR and alpha acetylcholine receptor expression profiles (as seen in myasthenia gravis) - the therapeutic effects of estrogen may be diminished or even contraindicated. Conversely, prophylactic estrogen pretreatment may offer protective benefits against acute inflammatory insults. Indeed, administration of pregnancy levels of estrogen does ameliorate the effects of acute inflammatory illnesses, as does the use of ER agonists, and pre-menopausal women exhibit lower risk of severity with respect to acute respiratory illnesses (170). Notably, estrogen receptor subtype expression patterns change with age (173), which may partly explain the poorer outcomes frequently observed in women over the age of 60–65 undergoing hormone replacement therapy (as discussed later). Thus, the immunological effects of estrogen appear to be context-dependent, influenced by a combination of factors including hormone levels, receptor subtype expression, cell type, activation state, local inflammatory conditions, and the experimental framework.

Nonetheless, the systemic impact of estrogen depletion during menopause has been widely documented (59), including declines in brain glucose metabolism, reductions in mitochondrial respiration, and loss of both white and grey matter volume (180, 181). These metabolic and inflammatory changes are further associated with increased β-amyloid accumulation and deterioration in neural function (180). Notably, elevated inflammasome protein levels have been observed in the hippocampus of aged rats (182) - a brain region essential for memory and emotional regulation. Hippocampal dysfunction, in turn, has been linked to the emergence of depressive and anxiety-like behaviors frequently seen during the menopausal transition. This is further compounded by a concurrent downregulation of ERβ in the dorsal raphe nucleus (DRN), where ERβ is a heavily represented estrogen receptor subtype. Its reduction corresponds with decreased numbers of tryptophan hydroxylase-immunoreactive (TPH-ir) neurons, suggesting impaired serotonergic tone, and has been associated with increased affective disturbances (182). Interestingly, an earlier age at menopause has also been linked to smaller hippocampal volume and diminished neural connectivity among carriers of the APOE4 allele - a known genetic risk factor for Alzheimer’s disease (183). This observation aligns with reports of more severe vasomotor symptoms and increased resistance to hormone-based treatments in APOE4-positive individuals (167, 184).

As previously mentioned, downregulation and/or desensitization of ER-β in the hippocampus has been shown to produce a range of detrimental effects on hippocampal tissue integrity and function. Estrogen’s modulation of inflammation within the hippocampus, as well as its role in emotional regulation and cognitive consolidation, is largely dependent on ER-β signaling (185). Notably, periodic activation of ER-β using the selective agonist DPN has been shown to protect hippocampal neurons from ischemic cell death in reproductively senescent female rats (186). This neuroprotection is accompanied by a reduction in inflammasome activation and other inflammatory pathways, as well as decreased levels of IL-1β proteins in the hippocampus (59, 186). Moreover, increased levels of inflammasome complexes in the cerebrospinal fluid of postmenopausal women suggest that the decline in estrogen and ER-β expression contributes to the emergence of a pro-inflammatory state (187).

Within the hypothalamus, shifts in estrogen receptor density during menopause contribute to functional changes in kisspeptin/neurokinin B/dynorphin (KNDy) neurons. In postmenopausal women, these KNDy neurons exhibit somatic hypertrophy and increased expression of kisspeptin and neurokinin B (NKB) gene transcripts, reflecting altered neuroendocrine signaling associated with estrogen decline (188). ERβ expression noticeably decreases in the hypothalamus following estradiol (E2) withdrawal in the context of menopause (189). However, the precise relationship between this receptor loss and the structural remodeling of KNDy neurons - and the downstream cellular consequences - remains unclear. In particular, the influence of ERβ signaling on KNDy neuron excitability and firing dynamics is not fully understood. Nonetheless, the observation that ERβ agonism improves vasomotor symptom (VMS) outcomes during the menopausal transition suggests that ERβ plays a regulatory role in this circuitry, likely through modulation of hypothalamic neuropeptidergic activity (167, 190).

## Reproductive aging in context: integrative insights and downstream effects

Hot flashes, a hallmark of menopause for many women in the United States, are not universally experienced across cultures. For instance, significantly fewer women in Japan, Korea, and Southeast Asia report hot flashes, and women in Mexico’s Yucatan peninsula reportedly do not experience them at all (191). These differences may reflect variations in cultural interpretations, semantics, and lifestyle factors such as diet. Despite extensive research, the mechanisms underlying hot flashes have remained elusive. While estrogen clearly plays a role - evidenced by the effectiveness of hormone therapy in alleviating vasomotor symptoms - it is not the sole factor.

The thermoregulatory center within the preoptic area (POA) of the hypothalamus plays a central role in maintaining core body temperature within a narrow “thermoregulatory zone” (192, 193). When body temperature exceeds the upper threshold of this zone, heat dissipation responses such as sweating are activated; when it falls below the lower threshold, mechanisms such as shivering are triggered. During menopause, the decline in ovarian follicle number and resulting reduction in estradiol production have been implicated in the emergence of vasomotor symptoms (VMS), including hot flashes. However, circulating estrogen levels alone do not consistently predict the frequency or severity of these symptoms (194).

Interestingly, individuals in perimenopause may exhibit transiently elevated estradiol levels (195; 9). Although it remains unclear whether VMS can occur entirely independent of estrogen fluctuation, the prevailing evidence suggests that erratic estrogen dynamics, rather than absolute low levels, play a contributory role. In this context, heightened activity of kisspeptin/neurokinin B/dynorphin (KNDy) neurons in the arcuate nucleus - key regulators of GnRH secretion and thermoregulatory output - appears to be a key mediator of impaired thermoregulatory stability. The abrupt withdrawal of estrogen, rather than sustained hypoestrogenism, seems to be the more potent trigger of VMS.

This is supported by observations in clinical populations. Women with gonadal dysgenesis, who maintain chronically low estradiol levels, typically do not experience hot flashes unless they receive estrogen replacement followed by abrupt discontinuation (196). Similarly, those who undergo bilateral oophorectomy - resulting in a sudden drop in estrogen - report more severe and frequent hot flashes compared to women undergoing natural, gradual menopause (197, 198). Additionally, individuals with functional hypothalamic amenorrhea (FHA), a state of hypoestrogenism caused by disrupted central GnRH signaling, rarely report VMS (199). These patterns suggest that sudden changes in estrogen signaling, rather than low levels per se, disrupt thermoregulatory control via estrogen-sensitive neural circuits. Importantly, studies have found no consistent differences in serum estradiol levels between women who experience hot flashes and those who do not, reinforcing the idea that VMS are driven by neuronal adaptations to hormonal variability rather than absolute hormone concentrations (200).

Furthermore, hot flashes typically peak during the early stages of menopause and gradually diminish post-menopause, even when circulating estrogen levels remain low (198). In women who experience hot flashes, the thermoregulatory zone is narrower, making small increases in core body temperature sufficient to trigger these episodes. This narrowing could explain individual susceptibility to hot flashes. Research indicates that this sensitivity is heightened in some women, resulting in an exaggerated thermoregulatory response to minor temperature fluctuations (201, 202).

Thus, the earliest step in the progression toward ovarian insufficiency - and the foundation for later acute symptomatology - is a diminished ovarian reserve and/or diminished follicular development combined with diminished hypothalamic sensitivity to estrogen (203, 204). This blunted responsiveness to estrogen’s negative feedback on GnRH signaling is evidenced by considerable decreases in AMH and subsequent increases in gonadotropin levels (particularly FSH and LH) that occur 5–10 years prior to the final menstrual period, well before a significant decline in circulating estrogen is observed.

The decline in AMH in particular coincides with compensatory upregulation of gonadotropin receptor expression, particularly the FSH receptor (FSHR), in an attempt to amplify FSH-FSHR signaling and maintain follicular recruitment and maturation.

However, despite this compensatory upregulation of gonadotropin receptors, the ovary’s capacity to respond remains fundamentally impaired. The limitation lies not in FSH signaling itself, but in the severe depletion of viable follicles, driven by altered hormonal feedback and a multifactorial, age-related decline in the structural and functional integrity of the ovary. As the follicular pool diminishes by orders of magnitude, the efficacy of FSH signaling is compromised, and receptor upregulation alone becomes insufficient to sustain normal ovarian function.

Prolonged gonadotropin insufficiency at the ovarian level can further reduce estradiol production, thereby exacerbating the decline in follicular recruitment and maturation (46). Compounding this dysfunction is a disruption in GnRH pulsatility: while ARC KNDy neurons continue to drive continuous, basal GnRH secretion, the surge-generating capacity - primarily dependent on positive feedback via AVPV kisspeptin neurons - is progressively lost. This results in inconsistent or blunted LH surges that are only occasionally sufficient to induce ovulation, ultimately leading to complete loss of the LH surge and cessation of ovulatory cycles (205).

Furthermore, diminished interaction between gonadotropin-releasing hormone (GnRH) and norepinephrine (NE) signaling has been observed in aging animal models. Specifically, a significant reduction in NE levels within the medial preoptic area (MPA) is associated with low serum luteinizing hormone (LH) levels and the loss of reproductive cyclicity (9). This decline in NE appears to be linked to the nitration of tyrosine residues on tyrosine hydroxylase (TH) - the rate-limiting enzyme in catecholamine synthesis - within NE neurons projecting to the MPA. Importantly, these neurochemical changes occur in a time-dependent manner, and are thought to result from chronic exposure to estradiol over the lifespan. The sustained hormonal milieu, rather than acute estrogen fluctuations, may contribute to progressive impairments in NE-GnRH interactions that ultimately compromise hypothalamic control of the reproductive axis (9).

Activation of neurokinin B (NKB) receptors (NK3R) in the median preoptic nucleus (MnPO), driven by increased NKB release from KNDy neurons, triggers heat-dissipating responses resembling menopausal hot flashes (206). The MnPO and medial preoptic area (MPA) receive thermal input from warm-sensitive skin afferents and regulate thermoeffectors like vasodilation and cold-seeking behavior. Infusion of the NK3R agonist senktide into the MnPO causes a rapid, dose-dependent drop in core temperature, while systemic NK3R agonists elevate tail skin temperature and lower core temperature - effects amplified in hypoestrogenic states such as post-ovariectomy. Conversely, antagonizing NK1R, NK2R, and NK3R in the MnPO blocks these responses (206).

Neurokinin signaling, particularly via NK3R and NK1R, appears critical to vasomotor symptoms (VMS). Substance P, the endogenous NK1R ligand co-expressed with NKB in KNDy neurons, further implicates NK1R in thermoregulation and sleep. Central infusion of substance P disrupts sleep in mice, an effect reversed by NK1R antagonists. In humans, intravenous substance P worsens mood, delays REM onset, fragments sleep, and triggers hot flashes. These findings support NK1R antagonism as a therapeutic strategy for managing both hot flashes and sleep disturbances during menopause (207).

There is increasing interest in the role of serotonin in the pathophysiology of hot flashes. Certain serotonin receptors are involved in thermoregulatory control and, when activated, can induce either a drop or rise in core body temperature (208). The decline in estrogen levels during menopause has been linked to reductions in circulating serotonin and a compensatory upregulation of certain hypothalamic serotonin receptors (209, 210). Estradiol is generally thought to support serotonergic signaling by stimulating the activity of tryptophan hydroxylase (TPH), the rate-limiting enzyme in the biosynthesis of serotonin from tryptophan (211, 212). Moreover, E2 downregulates the expression of the serotonin transporter (SERT) gene and functions as a SERT antagonist, thereby enhancing serotonin persistence within synaptic and extracellular environments (213).

Studies have shown that blood serotonin (5-HT) levels are significantly reduced in both naturally and surgically menopausal women due to estrogen deficiency. However, treatment with oestriol, a form of estrogen, restores serotonin levels to normal (209, 210). Furthermore, estrogen treatment has been linked to increased urinary excretion of 5-hydroxyindoleacetic acid (5-HIAA), the primary serotonin metabolite, indicating increased serotonin turnover (214).

Beyond its role in increasing serotonin levels, estrogen also influences serotonin receptor function and distribution. In the presence of progesterone, higher estrogen levels upregulate estrogen receptor beta (ERβ) (215, 216). ERβ activation leads to the upregulation of 5-HT2A receptors, which are associated with vasoconstriction (217, 219). Interestingly, the expression of ER-β is increased in response to E2 in older animals (218).

Another serotonin receptor, 5-HT1B, mediates vasodilation, and its activity is not uncoupled by E2 (unlike 5-HT1A receptors). Under normal physiological conditions, the vasoconstrictive actions of 5-HT2A receptors balance the vasodilatory effects of 5-HT1B receptors (220, 221). During menopause, an imbalance in estrogen and progesterone may result in inadequate compensatory activity of 5-HT2A receptors, leading to unopposed vasodilation and contributing to vasomotor symptoms such as hot flashes and night sweats (215). Additionally, the loss of estrogen reduces the density of 5-HT2A receptors and overall serotonin activity, further impairing thermoregulation. This dysregulation of serotonin signaling could explain the aberrant temperature regulation observed during menopause (222). The central nervous system (CNS), where 5-HT2A receptors are critical for thermoregulatory control, plays a key role in these processes. Studies have shown that drugs targeting 5-HT2A receptors can restore normal temperature regulation following ovariectomy (222). As mentioned elsewhere, reductions in Er beta activity, as well as progesterone and progesterone receptor signaling were associated with diminished TRH activity within the hippocampus and amygdala. Additionally, E2 therapy works in part by correctively modulating these perturbations - providing further evidence of the implications of these receptor density shifts on serotonin activity and menopausal symptoms.

The nighttime prevalence of hot flashes and night sweats may also be linked to the nocturnal conversion of serotonin into melatonin, which reduces circulating serotonin levels during sleep (223). This drop in serotonin may exacerbate thermoregulatory instability, contributing to increased vasomotor symptoms at night. Together, these findings underscore the complex interplay between estrogen, serotonin, and thermoregulation during menopause.

Increasing estrogen (E2) levels are associated with an upregulation of 5-HT2A receptor density and binding in the brain, along with a temporary reduction in the sensitivity of 5-HT1A receptor signaling (220, 221). Conversely, activation of ERα receptors, when estrogen is present alongside reduced progesterone levels, leads to an increase in 5-HT1A receptor activity, which is known to reduce serotonin production (216, 217).

Under normal physiological conditions, 5-HT2A2 receptor activity elevates intracellular calcium (Ca²^+^) levels, subsequently activating protein kinase C (PKC) (224). PKC activation plays a pivotal role in modulating serotonin regulation by disrupting the functionality of 5-HT1A1 autoreceptors. Specifically, PKC uncouples 5-HT1A1 ​autoreceptors from their signaling pathways, diminishing their capacity to regulate serotonin signaling (225–227).

This uncoupling weakens the inhibitory feedback typically provided by 5-HT1A1 autoreceptors, which ordinarily act to limit serotonin production. Consequently, 5-HT2A receptor activation through PKC amplifies this effect, rendering 5-HT1A autoreceptors ineffective and leading to elevated serotonin levels (225–227). Estrogen (E2) exacerbates this process by directly inhibiting 5-HT1A receptor function, further boosting serotonin concentrations (228, 229). Deviations from this process, such as reduced 5-HT2A receptor activity, may result in a loss of regulatory balance, leading to unrestrained vasodilatory effects. These effects can manifest as hot flashes.

Inflammation, often associated with diminished ovarian capacity, has been linked to various health outcomes, but its role in vasomotor symptoms (VMS) remains underexplored. While most studies have investigated inflammatory markers during or after the onset of VMS (230–232), few have clarified whether inflammation precedes or follows VMS. This gap leaves unanswered the critical question of causality: do elevated inflammatory markers trigger VMS, or are they a consequence of it?

Longitudinal research has yet to clearly differentiate between incident and prevalent vasomotor symptoms (VMS), or to clarify the temporal relationship between systemic inflammation and the onset of VMS (233). For example, Gold et al. (234) reported that adjusted hazard ratios (aHRs) for frequent VMS were higher than for infrequent symptoms, with elevated high-sensitivity C-reactive protein (hs-CRP) levels - measured either at baseline or concurrently - associated with frequent incident VMS, but only among premenopausal women (233, 234). Findings from the Study of Women’s Health Across the Nation (SWAN) further suggest that this association is strongest during the premenopausal and early perimenopausal stages, implying a stage-specific relationship between inflammation and VMS development.

The elevated hs-CRP levels frequently observed during perimenopause may in part reflect the influence of estradiol on inflammatory markers. Both endogenous and exogenous estrogens have been shown to modulate CRP concentrations (235–240). For instance, Eldredge et al. (236) found that a one standard deviation increase in endogenous estradiol was associated with a twofold increase in the likelihood of having CRP levels above the sample median. Similarly, users of hormone therapy (HT) with elevated levels of estradiol and sex hormone-binding globulin (SHBG) were more likely to have increased CRP concentrations (236). These findings raise the possibility that elevated estradiol levels prior to menopause may signal a heightened vulnerability to the inflammatory consequences of the subsequent sharp hormonal decline.

In parallel, menopause is characterized by intensified inflammatory signaling, along with the accumulation of non-cytokine inflammatory mediators within the ovaries themselves. These processes contribute to progressive reproductive decline and may hasten ovarian aging. As ovarian function deteriorates, normal patterns of hormonal cycling become increasingly disrupted. This hormonal disarray, in turn, sets the stage for the acute symptomatic changes that frequently accompany the menopausal transition.

## Implications for clinical management and modulative interventions for reproductive aging

### An overview of current hormone replacement therapy options

Menopause Hormone Therapy (MHT) primarily relies on estrogen to alleviate menopausal symptoms and manage health risks associated with hormonal decline. For women who have undergone a hysterectomy, estrogen can be administered alone, while women with an intact uterus require the addition of a progestogen to reduce the risk of endometrial hyperplasia or cancer (241, 242). Different forms of estrogen are used in menopause hormone therapy (MHT), each with specific characteristics, uses, and metabolic effects. Estradiol (E2) is a commonly prescribed estrogen due to its chemical similarity to the natural estrogen produced by the ovaries. It is highly effective in relieving menopause symptoms such as hot flashes and night sweats, improving bone density, and lowering fracture risk. Estradiol is available in various formats, including oral tablets, transdermal patches, gels, sprays, topical creams, and injectable forms.

Oral estradiol is metabolized in the small intestine and liver, converting into estradiol, estrone, and estrone sulfate. This process results in blood levels of estrone sulfate that are 5 to 10 times higher than estradiol levels (243). Estrone sulfate serves as a reservoir for the production of estradiol and estrone in tissues due to its long plasma half-life and slow clearance, supporting once-daily dosing. However, oral estrogen is associated with an increased risk of venous thromboembolic events (VTEs), especially in individuals with an increased risk of VTE and cardiovascular disease (244).

In contrast, non-oral delivery methods such as transdermal patches and gels bypass the liver’s first-pass metabolism. These methods maintain a balanced estradiol-to-estrone ratio (approximately 1:1) and reduce the risk of related side effects, making them an appealing alternative for some individuals (245). Conjugated estrogens, such as those in Premarin, consist of a mixture of estrogen compounds, including estrone and equilin, derived from equine sources or synthesized. These are commonly used for systemic menopause symptoms and bone health and are administered as oral tablets or vaginal creams. The inclusion of estrone in conjugated estrogens contributes to their systemic effects and allows for a sustained release of active compounds, similar to oral estradiol’s metabolism.

Estriol (E3), a weaker, naturally occurring estrogen, is primarily used for localized menopause symptoms such as vaginal dryness and irritation. It is commonly available as vaginal creams or suppositories and occasionally in oral formulations. Due to its minimal systemic effects and lower risk of endometrial stimulation, estriol is considered a gentler option, particularly for women who do not require systemic estrogen therapy. A newer option, estetrol (E4), is a naturally occurring estrogen produced by the fetal liver enzymes during pregnancy (241, 242). Estetrol provides systemic relief while potentially carrying a lower risk of VTE compared to stronger estrogens like estradiol. It is typically administered as oral tablets and represents an emerging option in menopause management. Estrone (E1), another naturally occurring estrogen, is weaker than estradiol and becomes more dominant post-menopause as the ovaries reduce estradiol production. Estrone is often included in conjugated estrogen formulations but is less commonly used alone in therapy. Meanwhile, ethinyl estradiol, a synthetic estrogen primarily used in contraceptives, is rarely employed for menopause therapy due to its higher potency and increased risks of VTE and cardiovascular events. For a summary of the different types of estrogens available on the market, see **Table 1**.

**Table 1:** Comparison of estrogen types used in menopause hormone therapy (MHT), highlighting potency, primary uses, delivery forms, and associated risks.

Estrogen type	Potency	Primary use	Forms available	Risks/notes	References
Estradiol	Most Potent	Systemic therapy	Oral, transdermal, topical	Clot risk (oral); bioidentical option	(246, 247)
Conjugated Estrogens	Moderate	Systemic and localized therapy	Oral, creams	Clot Risk (oral); Mix of estrogens; equine-derived	(248)
Estriol	Weak	Localized therapy	Vaginal, oral	Minimal systemic effects	(249)
Estetrol	Moderate	Emerging option	Oral	Lower clotting risk potential	(241)
Estrone	Weak	Part of conjugated estrogens	Oral, creams	Less commonly used alone	(245)

The table illustrates the key differences among various types of estrogens used in menopause hormone therapy (MHT), focusing on their potency, primary therapeutic applications, available delivery methods, and associated risks. It highlights how each type of estrogen, such as estradiol, conjugated estrogens, estriol, estetrol, estrone, and ethinyl estradiol, is uniquely suited to different aspects of menopause management. The table also underscores the importance of choosing the appropriate estrogen type and delivery method based on individual needs, balancing efficacy with potential risks such as venous thromboembolic events (VTEs) or hepatic effects. This visual comparison aids in understanding how estrogens vary in their systemic and localized effects, metabolism, and clinical applications.

For women who have not had a hysterectomy, combining progestogen therapy with estrogen is crucial to prevent endometrial hyperplasia, a potential risk of estrogen-only treatment. Progestogens include natural progesterone, identical to the hormone produced in the body, and synthetic versions known as progestins. Common progestins used in menopause hormone therapy (MHT) are dydrogesterone, which mimics natural progesterone closely; norethisterone acetate (NETA); drospirenone; levonorgestrel (LNG); and medroxyprogesterone acetate (MPA) (245). Progesterone is typically available in oral forms, while certain progestins, such as NETA and LNG, are included in transdermal patches combined with estradiol. The LNG-impregnated intrauterine device (LNG-IUD) is another option, offering localized protection against endometrial hyperplasia for up to eight years while minimizing systemic side effects (250).

Current guidelines generally do not view cardiovascular disease (CVD) risk factors as an absolute contraindication to estrogen therapy, especially in women under 60 (251). However, there are clear circumstances where estrogen therapy is not recommended. Findings from the Women’s Health Initiative (WHI) trial highlighted that the long-term use of estrogen therapy may pose significant health risks, including an elevated likelihood of venous thromboembolism (VTE), breast cancer, stroke, and coronary artery disease, outweighing the potential benefits (252). Even the latest guidelines stress exercising caution, particularly for individuals at the highest risk for these conditions (251, 253). Nonetheless, Menopausal hormone therapy (MHT) continues to be a recommended option for managing moderate to severe vasomotor symptoms, such as hot flashes, when prescribed at the lowest effective dose and for the shortest period needed (254, 255). For women with an elevated risk of venous thromboembolism (VTE) or cardiovascular disease (CVD) - including those who smoke, have obesity, or live with diabetes - transdermal or other non-oral estrogen delivery methods are often advised. These routes avoid first-pass metabolism in the liver, which helps lower the associated thrombotic and cardiovascular risks (251, 255).

Safety concerns regarding MHT frequently focus on its possible association with cancer, particularly breast cancer. Evidence from the Women’s Health Initiative (WHI) trials suggested that the use of oral conjugated estrogen combined with medroxyprogesterone acetate was linked to an increased risk of breast cancer (252). In contrast, this association was not observed with estrogen-only therapy (256). However, the WHI population was limited to women aged 50 to 79, many of whom had prior experience with hormone therapy, and the study examined only a narrow range of treatment regimens. A separate meta-analysis by Benkhadra et al. (257), which focused solely on randomized controlled trials, found that MHT - particularly estrogen-progestogen combinations - was associated with reduced mortality when started before age 60 or within ten years of menopause. Even so, hormone therapy is still discouraged or contraindicated in women who have a personal history or high risk of breast cancer or cardiovascular disease (257).

For non-oral MHT regimens, including estrogen-only or estrogen plus progestogen therapies, large clinical trials assessing their influence on breast cancer risk are lacking. Observational studies suggest that progestogens such as progesterone and dydrogesterone may have a more favorable breast safety profile compared to norethisterone acetate (NETA) and MPA (258, 259), although evidence for drospirenone remains limited. Furthermore, the potential breast cancer risk associated with the levonorgestrel intrauterine device (LNG-IUD) used in combination with estrogen therapy in postmenopausal women is uncertain, with current data limited to observational studies that have methodological limitations (260).

### HRT and VMS - in practice

More recent literature has investigated short-term and low dose estrogens for the treatment of vasomotor symptoms. A double-blind, placebo-controlled trial was undertaken to investigate the efficacy of micro-dose transdermal estrogen for relief of hot flashes. Women used transdermal patches with low dose estrogen (0.023 mg/d 17β-estradiol and 0.0075 mg/day levonorgestrel), micro-dose estrogen (0.014 mg/day 17β-estradiol) or placebo. Women using the micro-dose estrogen had a significant reduction in hot flashes when compared to placebo with 41% of patients having a reduction in hot flashes of 75% or more (261). Research on the effectiveness of low-dose estradiol gel for treating hot flashes tested 0.1% estradiol gel at daily doses of 1.0 mg, 0.5 mg, and 0.25 mg. All doses significantly decreased the frequency of hot flashes, with higher doses showing greater effects (262). Despite these promising results, it is still unclear whether lower doses of estrogen offer a distinct advantage in terms of their side-effect profile when compared to standard dosing regimens (263).

Progestational agents have emerged as a viable alternative to estrogen for managing vasomotor symptoms (VMS). Megestrol acetate has shown notable effectiveness, as demonstrated in a randomized, placebo-controlled trial involving 97 women with a history of breast cancer and 66 men undergoing androgen deprivation therapy for prostate cancer. Participants taking megestrol acetate at 40 mg/day experienced an 85% reduction in hot flashes, compared to a 21% reduction in the placebo group (264). Many participants continued using megestrol acetate for up to three years, maintaining symptom relief (265). Furthermore, a lower dose of 20 mg/day has proven equally effective, offering a potential option with a reduced dosage (266).

Medroxyprogesterone acetate (MPA), available in both oral and intramuscular formulations, has shown effectiveness in managing vasomotor symptoms (VMS). Early placebo-controlled trials from the 1970s and 1980s demonstrated significant VMS relief with intramuscular depot medroxyprogesterone acetate (DMPA) (267, 268). A comparative study of DMPA and megestrol acetate in postmenopausal women with a history of breast cancer reported equivalent reductions in hot flashes, averaging 86% in both groups (269). Observational studies have corroborated these findings, noting up to a 90% reduction in hot flashes with DMPA (270). Additionally, randomized trials have shown DMPA to be as effective as conjugated equine estrogen in treating VMS in women who have undergone hysterectomy or oophorectomy (271). Furthermore, a randomized controlled trial comparing venlafaxine to DMPA for VMS management demonstrated greater efficacy with DMPA. Women receiving a single 400 mg intramuscular dose of DMPA experienced a 79% reduction in hot flash scores, compared to a 55% reduction in the venlafaxine group. Notably, DMPA also had fewer short-term side effects compared to venlafaxine (272).

The efficacy of progesterone creams in managing hot flashes remains inconclusive, with results varying across studies. One placebo-controlled trial found an 83% reduction in hot flashes among women using progesterone cream, compared to 19% in the placebo group (273). However, a larger randomized trial involving 223 women found no significant difference between progesterone cream and placebo (274). Importantly, the type of progesterone used in these creams may influence their effectiveness, as variations in formulation could impact absorption and biological activity. Further research is needed to clarify the role of progesterone creams and whether specific formulations offer greater benefits.

Dehydroepiandrosterone (DHEA), a pro-androgen produced by the adrenal glands and liver, has been investigated for its potential to alleviate menopausal symptoms, given that its levels naturally decline with age (275, 276). Research conducted in 2000 revealed that supplementing with DHEA over six months led to a noticeable reduction in vasomotor symptoms among postmenopausal women (277). More recent findings from a pilot study of 22 participants highlighted a 50% reduction in hot flash severity and an improvement in quality of life following just four weeks of DHEA treatment (275). While these results are promising, further large-scale, randomized, placebo-controlled studies are required to establish the efficacy and safety of DHEA for menopausal symptom relief.

## Non-hormonal interventions

### SSRIs & SNRI

Non-hormonal options for managing vasomotor symptoms (VMS), such as SSRIs, SNRIs, and non-serotonergic drugs, provide alternatives to hormone-based treatments. Among the SNRIs, venlafaxine and desvenlafaxine have shown success in reducing hot flashes. Venlafaxine has been found to reduce the frequency of hot flashes by up to 61% at a dose of 75 mg/day, although higher doses do not offer additional improvements and can lead to side effects like nausea and sleep disruptions (278, 279). Desvenlafaxine, a derivative of venlafaxine, has demonstrated reductions of 60–64% at doses of 100–200 mg/day, with nausea as the primary dose-related adverse effect (280–282).

SSRIs, including paroxetine and citalopram, have also been effective for VMS. Paroxetine, administered at 10–25 mg/day, has shown reductions of 62–75% in hot flash severity, along with improvements in sleep quality, anxiety, and overall well-being. Side effects such as nausea, headaches, and insomnia have been reported but tend to be mild, particularly with lower doses (283, 284). Similarly, citalopram has shown reductions in hot flash frequency ranging from 37–58%, with its effects enhanced when used alongside hormone therapy (285–288).

However, it is important to note that a meta-analysis conducted by Rhodes et al. (289) found that approximately 79% of paroxetine’s therapeutic effect could be attributed to active placebo responses, with only about 21% representing a genuine pharmacological effect. Despite this, there remains a reasonable argument that paroxetine may still offer clinically meaningful benefits for a subset of individuals who respond favorably to its true drug action (289, 290).

Non-serotonergic treatments like gabapentin and pregabalin are additional options. Gabapentin, at a daily dose of 900 mg, has been shown to reduce hot flash frequency by 45–70%, with higher doses offering limited added benefit and mild side effects (291). Pregabalin, a newer drug, has demonstrated reductions of 65–71% at doses of 150–300 mg/day, though side effects like dizziness and drowsiness are mild and generally manageable (292, 293).

### Neurokinin receptor antagonist

Neurokinin 3 receptor (NK3R) antagonists are a newer class of medications that were originally developed in the 1980s for the treatment of schizophrenia, though that indication was later set aside. Today, these compounds are being repurposed for other clinical uses, with fezolinetant emerging as the first NK3R antagonist to be considered for FDA approval specifically for the treatment of moderate to severe vasomotor symptoms (VMS) related to menopause (294).

**Figure 4:**
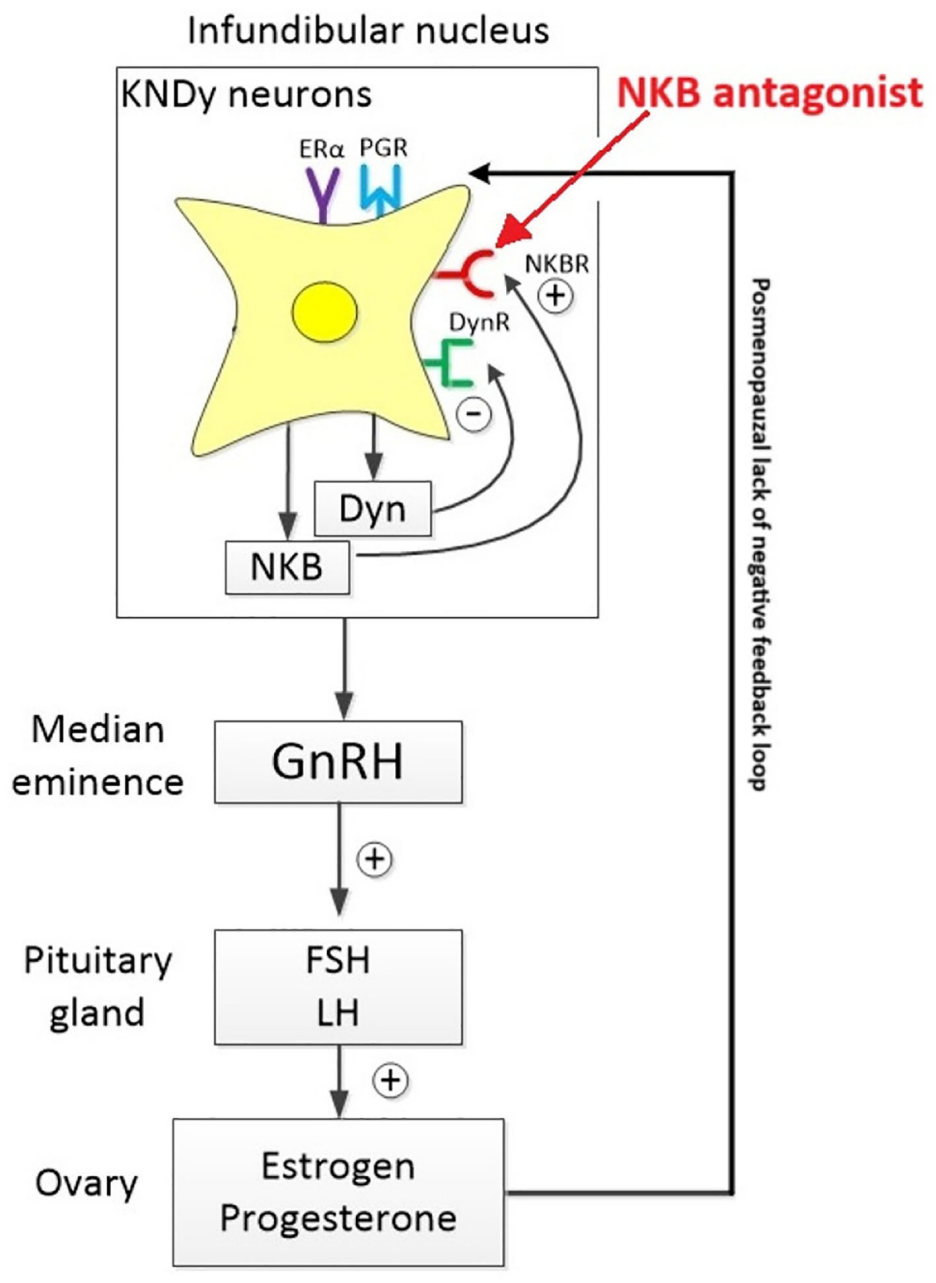
Neurokinin B (NKB), produced by KNDy neurons in the hypothalamus, activates GnRH neurons, thereby enhancing the release of gonadotropin-releasing hormone (GnRH). This stimulation triggers the secretion of follicle-stimulating hormone (FSH) and luteinizing hormone (LH), particularly under low estradiol conditions typical of menopause. In postmenopausal individuals, NKB signaling becomes overactive, contributing to disrupted thermoregulation and vasomotor symptoms. NKB receptor antagonists help mitigate this effect by dampening KNDy neuron hyperactivity, restoring balanced hypothalamic signaling and reducing inappropriate vasodilation. (Image reproduced from 206).

These drugs work by addressing the changes in brain signaling that occur with declining estrogen levels during menopause (**Figure 4**). A group of neurons in the hypothalamus known as KNDy neurons - named for their production of kisspeptin, neurokinin B, and dynorphin - play a key role in this process. Located in the preoptic area of the hypothalamus, these neurons activate NK3R and help regulate body temperature. Research by Rance et al. (295) showed that in postmenopausal women, where estrogen levels are greatly reduced, these neurons become enlarged and show increased expression of the neurokinin B gene. This suggests a strong link between estrogen withdrawal and heightened neurokinin B signaling. Since KNDy neurons project to the thermoregulatory center in the brain, their increased activity is thought to contribute to the onset of hot flashes. By blocking neurokinin B from binding to NK3 receptors, fezolinetant reduces the excessive signaling in this pathway and helps lessen hot flash symptoms.

Results from year-long international phase 3 clinical trials have evaluated the safety and effectiveness of two daily oral doses of fezolinetant - 30 mg and 45 mg - for treating VMS (294, 296). The SKYLIGHT trial series, which included randomized, double-blind, placebo-controlled 12-week studies followed by a 40-week safety extension, provided additional evidence of the drug’s benefit. Participants who initially received a placebo were later re-randomized to receive one of the two active doses. The primary outcome was the reduction in frequency and severity of hot flashes. Secondary outcomes included improvements in sleep, measured by a validated questionnaire (PROMIS Sleep Disturbance Short Form 8b). After 12 weeks, those taking 30 mg of fezolinetant saw a drop in hot flash frequency from 10.7 to 4.5 episodes per day, while the 45 mg group saw a decrease from 10.4 to 4.1 episodes per day. This represented a 56% and 61% average reduction from baseline, respectively. The placebo group showed a smaller reduction of 35%. The severity of hot flashes also declined. These improvements were sustained through the 52-week mark. However, improvements in sleep disturbance were not statistically significant at week 12 for either dose when compared to placebo.

Other SKYLIGHT trials reported similar outcomes in terms of safety and efficacy (297, 298). Early studies using higher doses of NK3R antagonists (e.g., 90 mg) reported a higher rate of gastrointestinal side effects. The most commonly reported side effects of fezolinetant included headache, digestive issues like abdominal pain and diarrhea, insomnia, back pain, and elevated liver enzymes. The increase in liver enzymes, reported in 1%–6% of participants, was typically mild and reversed after stopping the medication (296, 299). Interestingly, differences in response have also been noted across populations; for instance, fezolinetant appeared less effective in Asian women enrolled in the MOONLIGHT 1 trial (300), suggesting that genetic or physiological factors may influence its efficacy.

In addition to fezolinetant, elinzanetant is gaining attention as a promising non-hormonal candidate for treating moderate to severe vasomotor symptoms (VMS) associated with menopause. Acting as a dual antagonist of neurokinin-1 and neurokinin-3 receptors (NK1R and NK3R), elinzanetant has received market access for this indication, although it is not yet commercially available. The multinational, multicenter, double-blind Phase III SWITCH-1 trial demonstrated that a daily 120 mg dose of elinzanetant significantly reduced both the frequency and severity of hot flushes within four weeks of treatment, with participants also reporting meaningful improvements in sleep and overall quality of life (301).

Additional support comes from the Phase III OASIS-1 and OASIS-2 trials, which evaluated the safety and efficacy of elinzanetant in postmenopausal women experiencing moderate to severe VMS (302, 303). These studies enrolled 396 and 400 women, respectively, aged 40–65 years, across 184 clinical sites in 15 countries. Participants in the treatment group received 120 mg of elinzanetant daily for 26 weeks, while those in the placebo group received a matched placebo for 12 weeks, followed by elinzanetant for the remaining 14 weeks. Both trials demonstrated consistent reductions in VMS symptoms without any reported cases of endometrial hyperplasia, endometrial cancer, or liver toxicity, supporting the drug’s favorable safety profile (302, 303).

A recent systematic review and meta-analysis encompassing ten controlled trials and 4,663 postmenopausal women compared the clinical performance of fezolinetant and elinzanetant in managing VMS. Both agents were effective, achieving ≥50% reductions in symptom frequency by weeks 4 and 12. However, greater efficacy was observed in participants receiving elinzanetant at doses above 100 mg, particularly in terms of VMS severity and menopause-specific quality of life improvements (304). Elinzanetant also produced noticeable symptom relief as early as weeks 2 and 8, indicating a faster onset of action compared to fezolinetant. This earlier response may be especially valuable for women seeking quicker restoration of daily function and well-being. While higher doses of both drugs were associated with more side effects, elinzanetant generally displayed a more favorable tolerability profile—particularly in terms of sleep disturbances and gastrointestinal issues. These findings underscore the need for individualized dosing strategies to optimize treatment outcomes while minimizing adverse effects.

## Nutraceuticals, herbs, and VMS

### Vitamin E and vasomotor symptoms

Vitamin E was first proposed as a treatment for hot flashes in the 1940s (305, 306). Despite its early promise, much of the research on vitamin E supplementation has failed to differentiate between various formulations, often assuming that all products yield similar effects. This oversight neglects the unique properties of mixed tocopherol/tocotrienol blends compared to single-isomer tocopherol supplements. Mixed formulations are known to provide stronger anti-inflammatory effects, which could enhance their potential for alleviating menopausal vasomotor symptoms (307, 308). The eight isomers of vitamin E - each with distinct yet complementary properties - may work together to reduce inflammation and oxidative stress, emphasizing the need for formulation-specific considerations.

Studies comparing mixed tocopherol preparations with alpha-tocopherol alone have demonstrated the superior efficacy of mixed formulations in lowering oxidative stress markers, such as superoxide dismutase (SOD) activity and inducible nitric oxide synthase (iNOS) expression in cellular models. While both formulations showed protective effects, the mixed preparations provided markedly better outcomes, likely due to the inclusion of gamma- and delta-tocopherols. These findings suggest that omitting key isomers in some commercial products may limit their therapeutic success. As such, future research should prioritize exploring the impact of specific formulations to optimize vitamin E’s antioxidant and anti-inflammatory potential. Nonetheless, even single-isomer preparations have continued to show promise in addressing menopause-related symptoms, warranting further investigation.

The role of vitamin E in managing vasomotor symptoms has been explored in several studies, with slightly varying methodologies and results. One of the earliest randomized, crossover clinical trials, conducted in 1998, included 120 women who alternated between four weeks of vitamin E supplementation (800 IU daily) and four weeks of placebo. The analysis revealed that vitamin E reduced the frequency of hot flashes by about one episode per day and achieved a statistically significant reduction compared to the placebo. However, participants did not express a preference for vitamin E over placebo by the study’s conclusion (309). Another study by Cancelo Hidalgo et al. (310) demonstrated that a supplement combining vitamin E with isoflavones and primrose oil alleviates hot flashes and insomnia, though it remains unclear whether vitamin E alone would have produced similar results in this population. Additional research has also indicated potential benefits of vitamin E at lower doses. Ziaei et al. (311) observed decreases in the severity and frequency of hot flashes with a daily dose of 400 IU. Similarly, Ataei-Almanghadim et al. (312) noted improvements in hot flashes using 400 IU of vitamin E combined with curcumin.

More work should be done in the way of standardizing doses and ascertain the effectiveness of different doses for different patients. Further, studies should in the future assess the effects of mixed vitamin E in the way of managing VMS symptoms, relative to single isomer vitamin E.

### Isoflavones & lignins

It has been reported that only 10%–20% of women in Asia experience hot flashes. This is significantly less than the 80%–90% prevalence in the United States (191). The theory for this difference is the predominance of soybeans and soy-based dishes in the Asian diet. Soy is a potent source of phytoestrogens, which are naturally occurring compounds with both estrogenic and anti-estrogenic properties (313). Phytoestrogens can be classified into two main classes: isoflavones and lignans. Recently, there has been interest in phytoestrogens as treatment for hot flashes. Soy is a common source of isoflavones and has been studied as a treatment for hot flashes. In a randomized, double-blind, placebo-controlled trial, 91 perimenopausal women were given isoflavone-rich soy protein, isoflavone-poor soy protein, or whey protein. After 12 weeks there were no significant differences in hot flash frequency or severity between the 3 groups (314). A meta-analysis reviewed 11 trials of soy isoflavone extracts for treatment of hot flashes and overall the data was not positive (315). Soy phytoestrogens were also explored for the treatment of hot flashes in breast cancer patients. One double-blind clinical trial involved 177 breast cancer survivors who received either soy tablets or placebo. After 4 weeks there was no difference in hot flash frequency between groups (316). Further clinical trials of soy phytoestrogens in breast cancer patients also showed similar negative results (317). It is worth noting, however, that in some pharmacological trials, noticeable therapeutic effects typically take more than four weeks to emerge (318). Therefore, it remains plausible that long-term consumption of these compounds may yield more pronounced outcomes as treatment duration increases.

Lignan is another form of phytoestrogen, which is found in whole grains, legumes, vegetables, and in especially high concentrations in flaxseed (313). An increasing amount of research is currently being undertaken on flaxseed as treatment for hot flashes. A pilot trial involved 30 women who received 40 g of crushed flaxseed daily. After 6 weeks of therapy there was a 50% reduction in hot flash frequency (319).

A double-blind, randomized controlled trial involving 87 women investigated the effects of dietary flaxseed, soy, and wheat on menopausal symptoms. Participants consumed muffins containing 25 g of either flaxseed, soy flour, or wheat over a 16-week period. While neither flaxseed nor soy significantly impacted hot flash frequency, those in the flaxseed group reported a meaningful reduction in the severity of their hot flashes (320). Similarly, a Cochrane meta-analysis reviewing 30 clinical trials concluded that there was insufficient evidence to support the use of phytoestrogens in reducing the frequency or severity of hot flashes (321). However, earlier reviews, such as that by Howes et al. (322), suggested that isoflavone therapy may provide a modest reduction in hot flash occurrence. More recently, a meta-analysis by Chen et al. (323) found that isoflavones remained effective in reducing hot flash symptoms even after accounting for placebo effects, lending additional support to previous positive findings (324, 325). Still, the variability across studies - including differences in isoflavone composition, dosage, treatment duration, and outcome measures - makes it difficult to draw definitive conclusions. Nevertheless, given their favorable safety profile and broader health benefits, isoflavones remain a promising option for further exploration in the management of menopausal symptoms.

Red clover isoflavones, known for its isoflavone content, has also been investigated for its effects on menopausal symptoms. A Cochrane meta-analysis evaluating eight trials (ten comparisons) found that red clover significantly reduced the daily frequency of hot flashes in perimenopausal and postmenopausal women compared to placebo (326).

### Black cohosh

Black cohosh, a plant native to North America, initially showed promise in early research for reducing hot flashes (317). However, later studies have yielded variable outcomes. For instance, a crossover trial found no significant difference between black cohosh and placebo in relieving symptoms (327). Similarly, a randomized trial comparing black cohosh, red clover, and hormone therapy observed that neither herb outperformed placebo, although black cohosh reduced hot flashes by 34%, a result similar to placebo (328).

### Evening primrose oil (*Oenothera biennis*)

Evening primrose oil (Oenothera biennis), extracted from its seeds and rich in linoleic acid, has been evaluated as a potential treatment for hot flashes. In a randomized, double-blind, placebo-controlled study, researchers assessed the effects of 2,000 mg of evening primrose oil combined with 20 mg of vitamin E, taken twice daily for 24 weeks, on women experiencing more than three hot flashes per day. The study did not find significant benefits over placebo, and its results were limited by a small sample size and high dropout rates, with only 35 participants completing the trial (329). Conversely, other studies, such as one by Cancelo Hidalgo et al. (310), reported improvements in hot flashes and insomnia using a combination supplement that included evening primrose oil, isoflavones, and vitamin E. These inconsistent findings highlight the potential influence of factors such as dosing, formulation, and the inclusion of other compounds in determining efficacy.

### Dong quai (Angelica sinensis)

Dong quai (*Angelica sinensis*) is a Chinese herb which is traditionally used to treat menopausal symptoms. A randomized controlled trial in 71 women demonstrated no difference in hot flashes between dong quai treatment and placebo groups (330). Use of dong quai alone can be criticized because traditional Chinese practitioners never prescribe dong quai alone. Typically, it is used in conjunction with four or more other herbs as a polyherbal formulation. Nonetheless, over the counter single herb formulations are likely no more effective than placebo (330).

### Ginseng (Panax ginseng),

Ginseng (*Panax ginseng*), a widely used herbal supplement in the United States, is often sought after for its restorative properties, particularly for addressing fatigue and exhaustion. A randomized double-blind trial evaluated the effects of ginseng extract on vasomotor symptoms and quality of life in postmenopausal women. While the study found no significant reduction in hot flashes compared to the placebo, there was a slight improvement in overall symptom relief (331). Contrasting these findings, a more recent systematic review analyzed 15 randomized controlled trials involving 1,256 menopausal women and demonstrated that ginseng can alleviate menopausal symptoms, including hot flashes, while also enhancing quality of life (332).

### Wild yam (Dioscorea villosa)

Wild yam (*Dioscorea villosa*), often marketed as a natural progesterone cream due to its phytosterol content, has been promoted for managing menopausal symptoms like hot flashes. However, the diosgenin content in wild yam is relatively low and cannot be converted to progesterone *in vivo*. A double-blind, placebo-controlled crossover study by Komesaroff et al. (333) found no significant difference in symptom relief between wild yam cream and placebo.

In contrast, other studies have reported positive results for wild yam-based creams. For example, a study evaluating a wild yam salve found significant improvements in vasomotor symptoms, including hot flashes and night sweats, after three weeks of use (334). Additionally, research by Park et al. (335) demonstrated that wild yam extract at high concentrations induced progesterone receptor (PR) and Presenilin 2 (pS2) mRNA expression in MCF-7 cells within 24 hours, suggesting potential hormonal activity.

Further supporting its potential, a dietary intervention study by Wu et al. (336) revealed that replacing two-thirds of dietary staples with yam for 30 days resulted in a 26% increase in serum estrone, a near-significant 27% rise in estradiol, and a 9.5% increase in SHBG, alongside reductions in genotoxic estrogen metabolites. These hormonal shifts may help alleviate menopausal symptoms.

While evidence for wild yam products remains mixed, findings from both topical and dietary studies indicate potential benefits for managing menopausal symptoms, emphasizing the need for additional robust research to validate their efficacy and clarify mechanisms.

### Fenugreek (Trigonella foenum-graecum)

Fenugreek (*Trigonella foenum-graecum*) extract has been studied for its potential to alleviate perimenopausal symptoms and support hormonal balance. A randomized, double-blind, placebo-controlled trial by Khanna et al. (337) demonstrated that supplementation with a standardized fenugreek extract (FHE) significantly reduced vasomotor symptoms and depression in perimenopausal women without adverse effects. Hormonal analysis revealed notable improvements, including increases in serum estradiol (18.9%), free testosterone (38.2%), and progesterone (19.9%), along with reductions in FSH (38.2%) and SHBG (21.1%), contributing to a more balanced hormonal profile. These findings are supported by additional research reporting similar benefits of fenugreek extract in improving menopausal symptoms and hormonal regulation (338–340). Collectively, these studies suggest that fenugreek extract may be a safe and effective non-hormonal option for managing perimenopausal discomforts.

### Estro G-100

EstroG-100^®^ is a standardized herbal formulation derived from three plants: *Cynanchum wilfordii*, *Phlomis umbrosa*, and *Angelica gigas*. Clinical investigations suggest it may provide relief for a range of menopausal symptoms.

In a randomized, double-blind, placebo-controlled trial conducted by Chang et al. (341), 64 women across premenopausal, perimenopausal, and postmenopausal stages - spanning White Hispanic, White non-Hispanic, and African American backgrounds - were evaluated over a 12-week period. The group receiving EstroG-100^®^ showed significant improvements in multiple symptoms, including hot flashes, paresthesia, insomnia, nervousness, low mood, dizziness, fatigue, and joint pain, compared to the placebo group.

Kim et al. (342) carried out a larger multicenter, randomized, double-blind, placebo-controlled study involving women aged 40 to 70 experiencing menopausal complaints. After 12 weeks of treatment, total scores on a modified Kupperman Menopausal Index (KMI) significantly improved in the EstroG-100^®^ group relative to placebo. Specific symptoms such as paresthesia, emotional instability, dizziness, fatigue, joint and muscle pain, and formication were notably alleviated. Importantly, there were no significant changes in serum estradiol (E2), follicle-stimulating hormone (FSH), endometrial thickness, or body mass index (BMI), supporting the extract’s safety profile.

A separate double-blind, placebo-controlled study by Farzaneh et al. (343) focused on postmenopausal women reporting hot flashes. Participants were randomized to receive either EstroG-100^®^ or a placebo for 12 weeks. By week six, those in the treatment group reported significantly fewer hot flashes, while no adverse effects were noted.

Snigdha et al. (344) examined a novel combination therapy of EstroG-100^®^ (514 mg) with gamma-aminobutyric acid (GABA, 50 mg) in a randomized controlled trial. Assessments using validated symptom questionnaires revealed improvements as early as day three in mood, stress, and headache frequency. Between weeks two and five, participants receiving the combination treatment reported reductions in vasomotor symptoms (by day 7), improved sleep quality (by week 5), and enhancements in mood and sexual function.

Finally, a prospective, single-arm interventional study conducted by Kirubamani et al. (345) used the Menopause Rating Scale-11 and the Menopause Symptoms Treatment Satisfaction Questionnaire (MS-TSQ) to assess outcomes in participants taking EstroG-100^®^. Significant improvements in somatic, urogenital, and psychological symptoms were observed at six weeks (P = 0.001), with even more pronounced benefits after 12 weeks (P < 0.0001). The majority of participants (96.5%) reported satisfaction with the treatment.

### Rheum rhaponticum

Rheum rhaponticum, commonly known as rhubarb, has long been used in Germany to manage menopausal symptoms. Its therapeutic potential is largely attributed to rhaponticin, a primary active compound found in its root. A standardized root extract, ERr 731^®^, has been clinically investigated for its efficacy and safety in alleviating climacteric symptoms in peri- and postmenopausal women. What makes ERr 731^®^ particularly noteworthy is its selective action on estrogen receptor beta (ER-β), avoiding stimulation of estrogen receptor alpha (ER-α). This selectivity minimizes the risks often associated with conventional hormone therapy, especially estrogen-sensitive tissue proliferation.

Preclinical research using ovariectomized animal models has demonstrated that ERr 731^®^ does not induce uterine or endometrial proliferation - effects commonly observed with direct estrogen supplementation (190). Additional long-term toxicity studies confirmed a high safety threshold, reporting no adverse effects at doses up to 1000 mg/kg/day (190).

Post-marketing data further support its safety profile. From 1993 to mid-2014, approximately 140 million daily doses were distributed in Germany, with only 124 adverse event (AE) reports, primarily consisting of hypersensitivity reactions (n=74) and gastrointestinal complaints (n=47). In North America, 79 consumer complaints were recorded between 2009 and 2014 following the sale of 13 million tablets, with most reports citing digestive discomfort (n=23) or perceived lack of efficacy (n=22). Interestingly, no complaints were registered in South Africa from the product’s market entry in 2011 through mid-2014 (346).

A recent meta-analysis - the first of its kind - by Dubey et al. (190) found that ERr 731^®^ significantly reduces menopausal symptoms. Although the possibility of reporting bias exists, the observed treatment effect appears too strong to be solely attributed to such bias, particularly given that placebo responses in menopause trials rarely exceed 40–50% efficacy (347). Additionally, outcomes from non-randomized observational studies have been consistent with those reported in randomized controlled trials (190, 346). These findings also align with experimental evidence supporting the modulatory effects of ER-β agonists in preclinical models (348).

## Conclusion

Reproductive aging is a multifaceted biological process that extends beyond ovarian senescence, encompassing a complex interplay of neuroendocrine, metabolic, and immunological adaptations. At its foundation, menopause results from the depletion of ovarian follicles and the subsequent decline in estradiol and progesterone levels. This decline reflects the cumulative effect of decades of estrogen-mediated signaling within the hypothalamus, ultimately destabilizing the tightly regulated feedback loops of the hypothalamic-pituitary-gonadal (HPG) axis.

Importantly, this hormonal transition is neither abrupt nor uniform. Instead, it initiates a gradual reorganization of signaling within key hypothalamic networks - most notably those involving kisspeptin, neurokinin B (NKB), GABA, and glutamate. These neurochemical shifts, particularly within KNDy (kisspeptin/NKB/dynorphin) neurons, disrupt gonadotropin-releasing hormone (GnRH) pulsatility and contribute to hallmark menopausal symptoms such as vasomotor instability, irregular ovulatory cycles, and altered reproductive hormone secretion.

The physiological cascade of menopause is further compounded by systemic inflammation, mitochondrial dysfunction, oxidative stress, and impaired DNA repair, all of which accelerate ovarian and neural aging. Estrogen receptor (ER) signaling - especially the divergent actions and regional expression of ERα and ERβ - plays a critical role in modulating these transitions. The tissue- and receptor-specific responses to declining estradiol levels help explain individual differences in symptom patterns, therapeutic outcomes, and long-term health risks.

Moreover, genetic, cultural, and environmental influences - including dietary habits, APOE genotype, and exposure to endocrine-disrupting chemicals - modulate the onset, severity, and nature of menopausal symptoms. These variations highlight the need for personalized approaches to treatment. Novel therapies targeting specific neural circuits, such as neurokinin-3 receptor antagonists and ERβ-selective modulators, show promise as alternatives to traditional hormone therapy, particularly for individuals in whom estrogen replacement is contraindicated or ineffective.

Altogether, menopause should be understood not as a single endocrine event but as a broader process of neuroimmune remodeling. Advancing our mechanistic understanding of the molecular, cellular, and circuit-level alterations that drive reproductive aging is essential for improving diagnostic precision, guiding therapeutic innovation, and ultimately enhancing quality of life for aging women. Future research must continue to explore the interaction between central and peripheral mechanisms - while accounting for genetic and environmental variability - to fully unravel the biology of menopause and inform next-generation interventions.
